# Supplementary material for: Tumor-activated in situ synthesis of single-atom catalysts for O2-independent photodynamic therapy based on water-splitting
Source: Nat Commun. 2024 Apr 6;15:2954. doi: 10.1038/s41467-024-46987-1 (PMC11258260; doi:10.1038/s41467-024-46987-1)
Supplement: Supplementary file 1 — Supplementary Information [file 41467_2024_46987_MOESM1_ESM.pdf]

Supplementary Information

Tumor-activated in situ synthesis of single-atom catalysts for O_2-independent photodynamic therapy based on water-splitting

Yiyan Yin[1], Xiyang Ge[1], Jin Ouyang[2] and Na Na[1,*]

1. Key Laboratory of Radiopharmaceuticals, Ministry of Education, College of Chemistry,

Beijing Normal University,

Beijing, 100875, China.

*E-mail: nana@bnu.edu.cn

2. Department of Chemistry, College of Arts and Sciences, Beijing Normal University at Zhuhai,

Zhuhai, 519087, China.

Table of Contents

1. Supplementary method……………………………..………………………………………………..……1

2. Supplementary figures………………………….…………………………………...………………3

3. Supplementary References……………………………………………...……………………………..32

Supplementary method

Chemicals. Melamine, 1,3-Diphenylisobenzofuran (DPBF), $NaBiO_3$, p-Phthalic acid (PTA), deferoxamine (DFO), and 3,3',5,5'-tetramethylbenzidine were obtained from Macklin reagent (Shanghai, China). $KMnO_4$ of analytical grade was purchased from Beijing Chemicals (Beijing, China). 4-Morpholineethanesulfonic acid (MES), H_2O_2 (30%), and Linoleic acid (LA) were purchased from Innochem (Beijing, China). Glutathione (GSH, reduced form), N-Ethylmaleimide (NEM), PBS, Dulbecco's Modified Eagle's Medium (DMEM), and fetal bovine serums (FBS) were purchased from Sangon Biotechnology Co., LTD. (Shanghai, China). $[Ru(dpp)_3]Cl_2$ (Luminescent oxygen sensor) was purchased from Aladdin (Shanghai, China). 2',7'-Dichlorodihydrofuorescein diacetate (DCFH-DA), MDA kits, GSH kits, 3-[4,5-dimethylthiazolyl-2-]-2,5-diphenyltetraolium bromide (MTT), JC-1, propidium iodide (PI) and Calcein AM were purchased from Solarbio (Beijing, China). All other chemical reagents were of analytical grade and were used directly without further purification. Methanol of HPLC grade was purchased from Fisher Chemical (CA, USA). Ultrapure water (Mill-Q, Millipore, 18.2 MΩ) was used in all experiments.

Instruments. Transmission electron microscopy (TEM) measurements were carried out on a Talos F200S transmission electron microscope (Thermo Fisher Scientific). A scanning electron microscope (SEM, SU-8010, Hitachi) was used to determine the morphology of the as-prepared samples. Powder X-ray diffraction patterns were recorded by Shimadzu XRD-7000 with Cu Kα radiation (λ = 1.5418 Å). High-angle annular dark-field scanning transmission electron microscopy (HAADF-STEM) characterization was conducted on an FEI Themis Z. Atomic force microscopy (AFM) images were taken using a Bruker Dimension ICON. XPS data were obtained with a K-Alpha electron spectrometer from Thermo Scientific. The actual Mn content was determined by using inductively coupled plasma optical emission spectroscopy (ICP-OES, Agilent 5110). The ζ potentials of as-prepared samples were measured by a Malvern Nano ZS90. Fourier Transform infrared spectroscopy (FTIR) spectra were obtained on a Thermo Scientific Nicolet iS20. Raman spectra were obtained on a Horiba LabRAM HR Evolution with an excitation wavelength of 532 nm. Ultraviolet-visible spectroscopy (UV-vis) measurements were recorded on a Shimadzu UV-2450 spectrophotometer. Fluorescence measurements were carried out using a Shimadzu RF-6000 spectrofluorometer. EPR data of radicals were obtained with a Bruker A200 and data of C_3N_4-Mn were obtained with a Bruker EMXplus-6/1. MS analysis was performed using an LTQ XL linear ion trap mass spectrometer (Thermo Fisher Scientific). High-resolution mass spectrum (HRMS) data were recorded on an AB triple-TOF 5600 HRMS. Confocal laser scanning microscopy (CLSM) images of cells were performed using a Laser Scanning Confocal Microscope system (Nikon A1). Online monitoring of GSH concentration was carried out using a homemade ionization source reported before.[1] The X-ray absorption fine structure spectra were collected at the 1W1B station in the Beijing Synchrotron Radiation Facility (BSRF). The storage rings of the BSRF were operated at 2.5 GeV, with an average current of 250 mA. Using a Si(111) double-crystal monochromator, the data collection was collected in fluorescence mode using an ionization chamber. All spectra were collected under ambient conditions, and XAFS data were processed using the ATHENA module implemented in the IFEFFIT software packages according to standard procedures. The k^3-weighted EXAFS spectrum was obtained by subtracting the post-edge

background from the overall absorption and then normalized with respect to the edge-jump step. Subsequently, the k^3-weighted χ(k) data of the Mn K-edge were FT to real space (R space) using Kaiser-Bessel windows (dk = 1.0 $Å^{-1}$) to separate the EXAFS contributions from different coordination shells. The EXAFS Wavelet analysis was performed through the Fortran software packages[2].

Computational details. The spin-polarized density functional theory (DFT) calculations were carried out by using the Vienna Ab initio Simulation Package (VASP, 6.3.0).[3] Electron-ion interactions were described by using the projector-enhanced waves (PAW) potential.[4] The optimization of geometric structure and the electronic structure was calculated with the generalized gradient approximation (GGA) and Perdew-Burke-Ernzerhof (PBE) functions.[5] The band structure of C_3N_4 and C_3N_4-Mn were calculated by Heyd-Scuseria-Ernzerhof (HSE06) hybrid functional.[6] The cut-off energy of the plane wave basis set was taken to be 450 eV. For Brillouin zone sampling, the k-points using the Monkhorst-Pack grids of 1 x 1 x 1 and 5 x 5 x 1 were adopted for geometry optimization and density of states, respectively. The convergence threshold for the total energy and forces were set to be 1 E-05 eV and -0.01 eV $Å^{-1}$, respectively. The vacuum layer of C_3N_4 and C_3N_4-Mn was set to 20 Å to avoid the interlayer interactions. To implicitly incorporate the solvation effect, the linearized Poisson Boltzmann model (PBM) was utilized to represent the double layer. This approach has been incorporated into VASPsol and the compensatory charge was represented by adjusting the Debye screening length to 3.04 Å. The relative permittivity was fixed at 78.4 to simulate an aqueous environment.[7] The binding energy of Mn^{2+} on the C_3N_4 was defined as: $E = E(C_3N_4\text{-Mn}) - E(C_3N_4) - E(Mn^{2+})$, where $E(C_3N_4\text{-Mn})$, $E(C_3N_4)$, and $E(Mn^{2+})$ were the total energy of C_3N_4-Mn, C_3N_4, and relaved free Mn^{2+}, respectively. The charge density difference (CDD) map due to the adsorption of Mn was obtained as: $\Delta\rho = \rho(C_3N_4\text{-Mn}) - \rho(C_3N_4) - \rho(Mn^{2+})$, where $\rho(C_3N_4\text{-Mn})$, $\rho(C_3N_4)$, and $\rho(Mn^{2+})$ were the electron density of C_3N_4-Mn, C_3N_4, and related free Mn^{2+}, respectively. The DFT energy change of each elementary step was defined by VASPsol, and the Gibbs free energy was calculated as $\Delta G = \Delta E + \Delta ZPE - T\Delta S$, where ΔE was the total energy difference obtained by DFT calculation, ΔZPE was the zero-point energy change and $T\Delta S$ represents the energy correction by entropy. T was set to 298.15 K and the pH-induced free energy change was not considered.

Supplementary figures

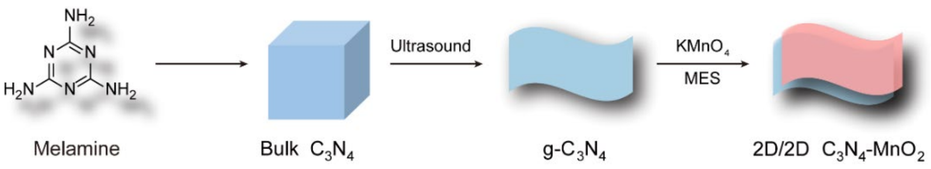

Supplementary Figure 1. The synthesis scheme of C_3N_4-MnO_2.

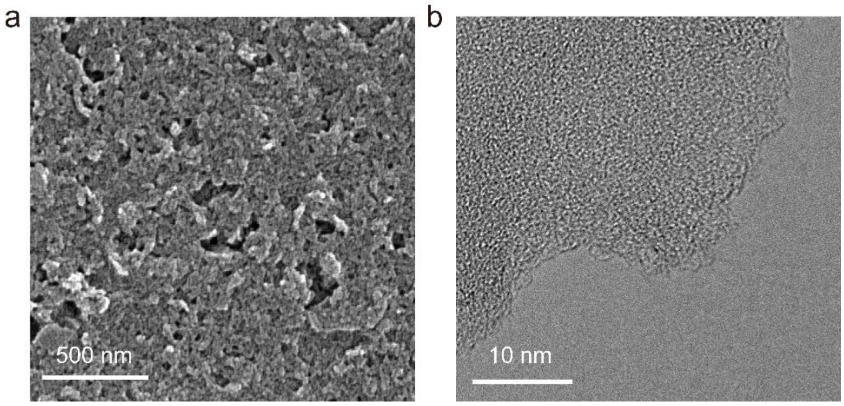

Supplementary Figure 2. a SEM and **b** TEM characterization of C_3N_4 nanosheets.

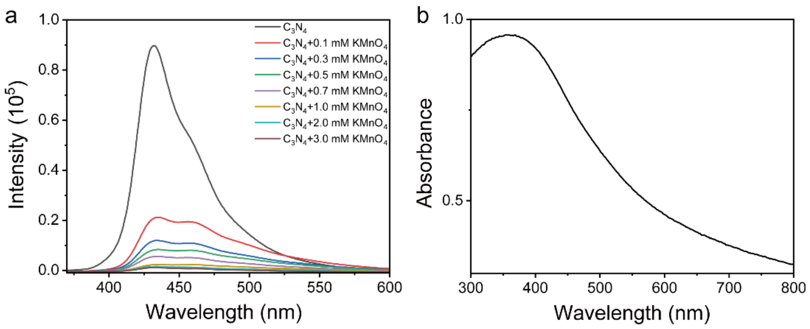

Supplementary Figure 3. a Fluorescence spectra of C_3N_4 nanosheet after MnO_2 deposited upon the reduction of $KMnO_4$ at different concentrations. The excitation wavelength was 312 nm. The experiment was repeated three times independently with similar results. **b** The UV-Vis absorption of MnO_2. c(g-C_3N_4) = 25 μg/mL, c(MnO_2) = 100 μg/mL.

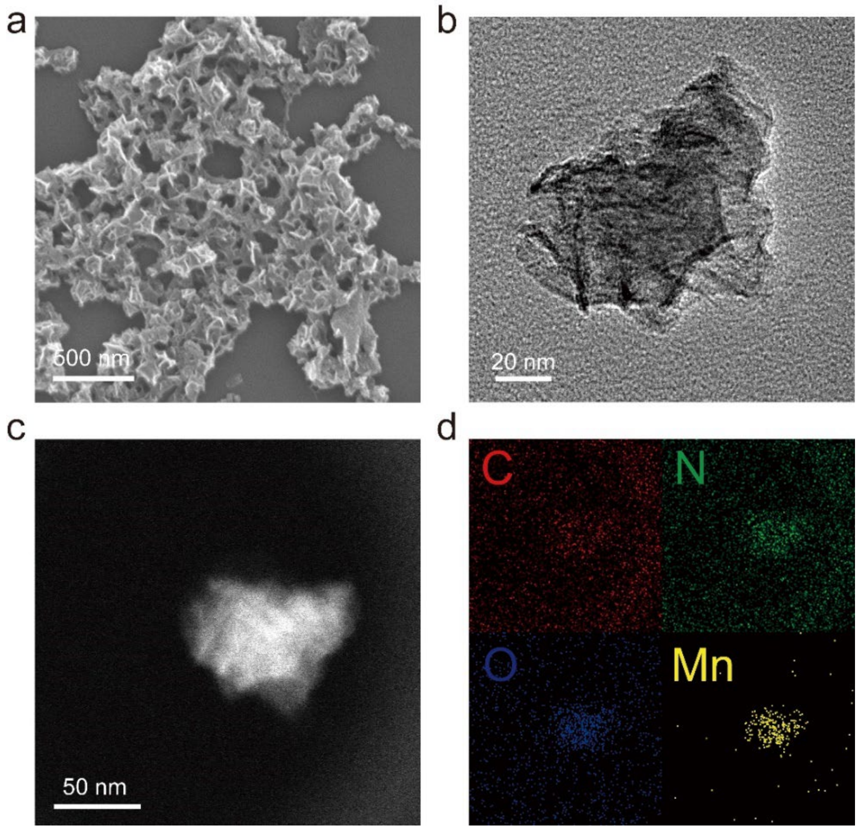

Supplementary Figure 4. a SEM and **b** TEM images and **c-d** EDX elemental mapping images of C_3N_4-MnO_2.

Firstly, the bulk C_3N_4 component was prepared via the calcination of melamine followed by the chemical oxidation by HNO_3. Thereafter, the bulk C_3N_4 component was exfoliated into the g-C_3N_4 nanosheets upon ultrasonication in water for 16 h (Supplementary Fig. 1). As characterized by Scanning Electron Microscopy (SEM) and Transmission Electron Microscopy (TEM) (Supplementary Fig. 2), the g-C_3N_4 nanosheets exhibited a layered 2D structure with a somewhat uniform size distribution. Subsequently, 2D/2D C_3N_4-MnO_2 nanocomposite was constructed by the reduction of $KMnO_4$ onto g-C_3N_4 nanosheets (Supplementary Fig. 1). The fluorescence (FL) intensity of C_3N_4 significantly decreased with the increase of $KMnO_4$ concentration till 1 mM (Supplementary Fig. 3). It can be observed that MnO_2 have a broad absorption band, which overlaps well with the fluorescence emission of the g-C_3N_4. Therefore, this FL quenching was induced by the absorption of excited electrons by MnO_2, simultaneously confirming the successful deposition of MnO_2 on g-C_3N_4 nanosheets.[8] Considering no remarkable FL change was observed when $KMnO_4$ concentration was higher than 1 mM, 1 mM $KMnO_4$ was adopted to synthesize C_3N_4-MnO_2. The growth of MnO_2 on g-C_3N_4 nanosheets was further validated by SEM characterizations, TEM images, and mapping images (Supplementary Fig. 4).

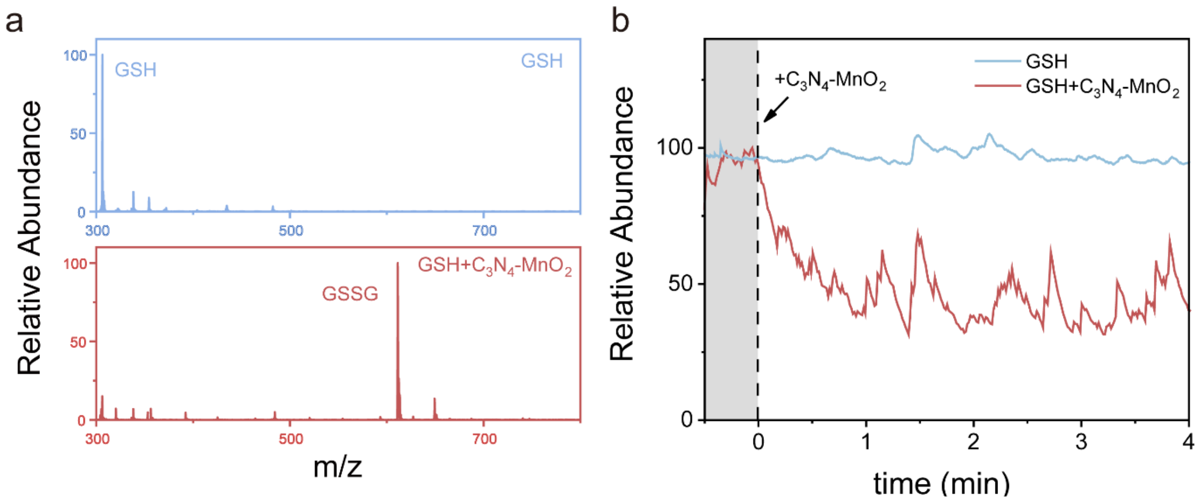

Supplementary Figure 5. a Mass spectra before and after the in situ synthesis of C_3N_4-Mn SACs by GSH. The experiment was repeated three times independently with similar results. **b** Real-time monitoring of GSH with or without C_3N_4-MnO_2 precursors.

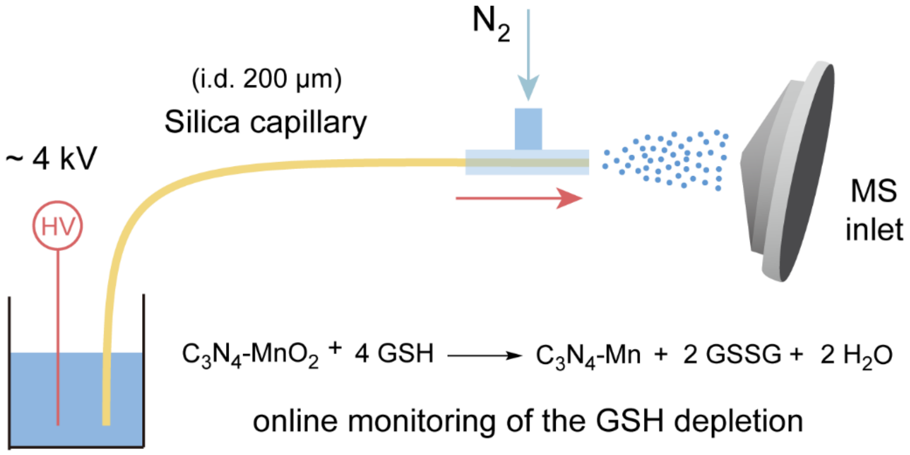

Supplementary Figure 6. Diagram of the online monitoring instrument for the GSH depletion by C_3N_4-MnO_2 nanocomposite.

The consumption of GSH was confirmed by mass spectrometry (MS) detections (Supplementary Fig. 5a), which exhibited the decreased $[GSH - H]^-$ (m/z 306) along with $[GSSG - H]^-$ (m/z 611) increasing. The extracted ion chromatograms (EICs) of online MS monitoring further displayed the reduction of C_3N_4-MnO_2 precursor by GSH (Supplementary Fig. 5b, 6).[9]

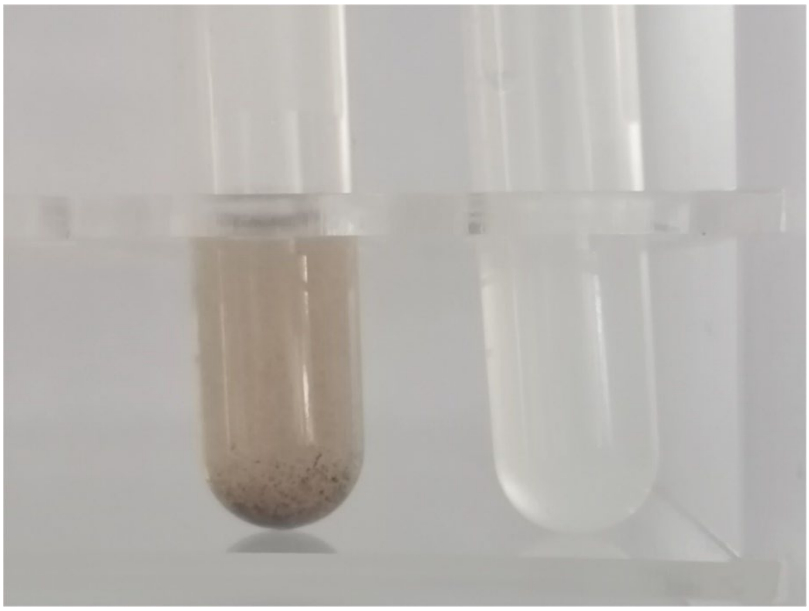

Supplementary Figure 7. Photo of C_3N_4-MnO_2 (left) and after exposure to GSH for 5 min (right). c(C_3N_4-MnO_2) = 100 μg/mL, c(GSH) = 10 mM.

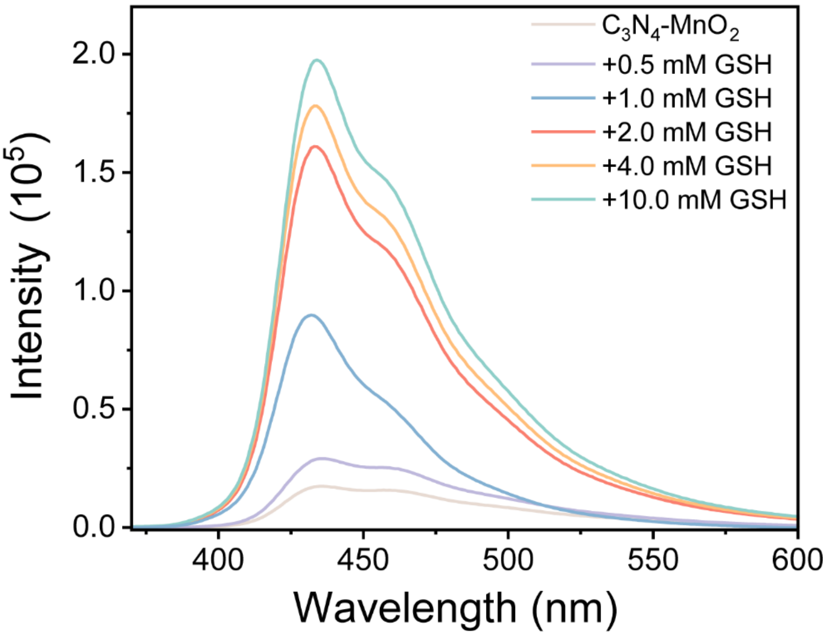

Supplementary Figure 8. Fluorescence emission spectra of C_3N_4-MnO_2 in the presence of different concentrations of GSH excited at 312 nm. The experiment was repeated twice independently with similar results. The concentration of C_3N_4-MnO_2 was 100 μg/mL.

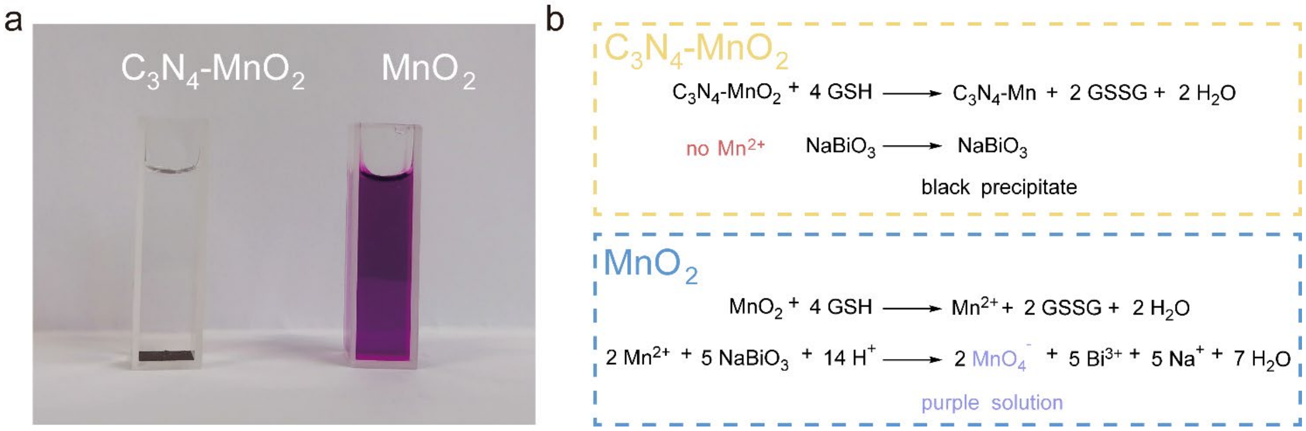

Supplementary Figure 9. Evaluation of the presence of free Mn^{2+} by $NaBiO_3$ after reacting with GSH. **a** Pictures of the C_3N_4-MnO_2 (Left) and MnO_2 (Right) after reacting with GSH. **b** Schematic representation of the reaction between C_3N_4-MnO_2 or MnO_2 and GSH respectively, as well as the detection of free Mn^{2+} by $NaBiO_3$.

As shown in Supplementary Fig. 9, free Mn^{2+} can be obtained upon the reaction between MnO_2 with GSH. The obtained Mn^{2+} would be converted into the purple MnO_4^- by the HNO_3-$NaBiO_3$ system, making the solution turn purple (right). Along with the reduction of C_3N_4-MnO_2 by GSH, Mn^{2+} was released and captured by the g-C_3N_4. Therefore, no free Mn^{2+} was left in the supernatant of the reacted solution. That's why the solution in the left cuvette is not purple.

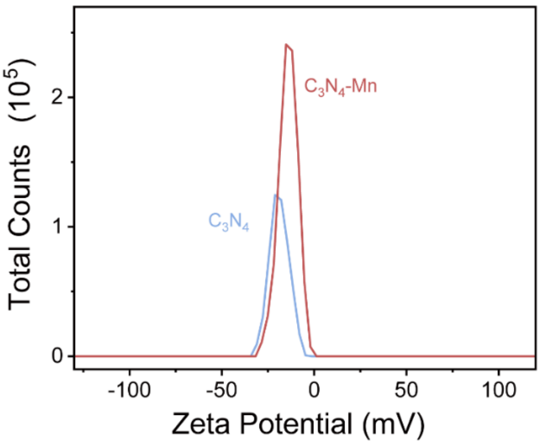

Supplementary Figure 10. ζ potential of the g-C_3N_4 and C_3N_4-Mn in water.

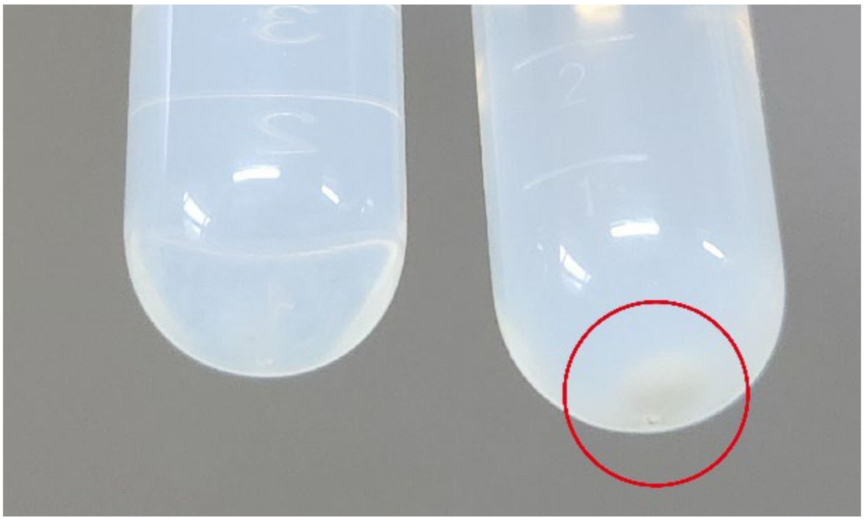

Supplementary Figure 11. The picture of C_3N_4 (left) and C_3N_4-Mn (right) standing for 24 h.

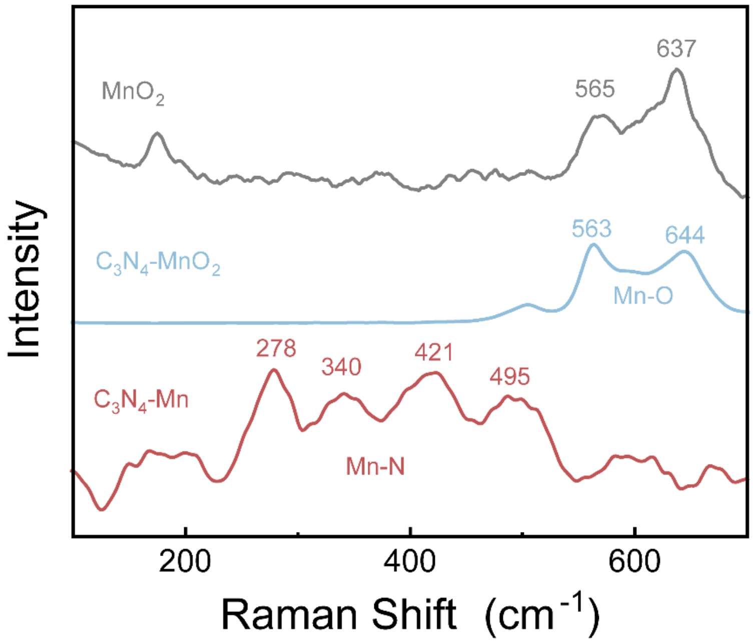

Supplementary Figure 12. Raman spectra of MnO_2, C_3N_4-MnO_2, and C_3N_4-Mn SACs.

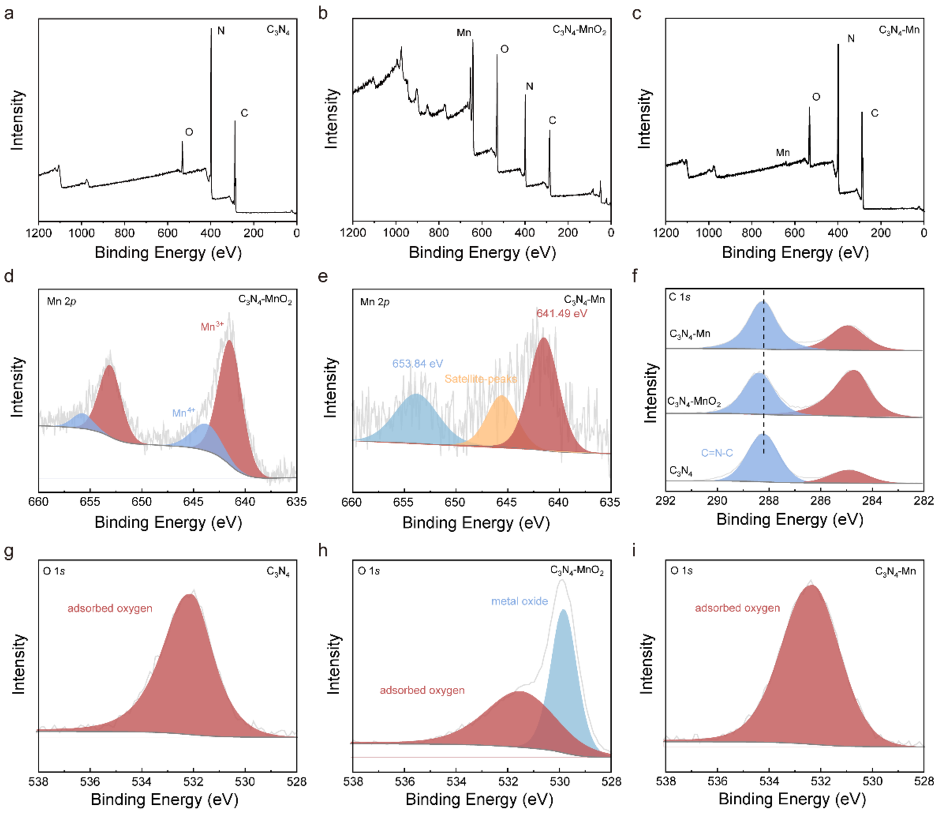

Supplementary Figure 13. XPS spectra of **a** g-C_3N_4, **b** C_3N_4-MnO_2, and **c** C_3N_4-Mn. Mn 2*p* spectra of **d** C_3N_4-MnO_2, **e** C_3N_4-Mn SACs, and **f** C 1*s* spectra of C_3N_4, C_3N_4-MnO_2, and C_3N_4-Mn, **g** O 1*s* spectra of C_3N_4, **h** C_3N_4-MnO_2, and **I** C_3N_4-Mn at high resolution.

As demonstrated in Supplementary Fig. 12, the bands of 565 and 637 cm^{-1} (MnO_2) are slightly shifted in the Raman spectra of C_3N_4-MnO_2, showing the significant interaction between C_3N_4 and MnO_2.[10] As shown in Supplementary Fig. 13, the peaks of Mn(III) (Mn 2$p_{1/2}$ at 653.1 eV and Mn 2$p_{3/2}$ at 641.5 eV)[11] and Mn(IV) (Mn 2$p_{1/2}$ at 655.7 eV and Mn 2$p_{3/2}$ at 643.8 eV)[12,13] were recorded in C_3N_4-MnO_2 (Supplementary Fig. 13d). Besides, with MnO_2 deposed on C_3N_4, the characteristic peaks of N-C_3 (400.5 eV), C−N=C (398.7 eV) as well as C−N=C (288.2 eV) in C_3N_4 (Supplementary Fig. 13f) shifted to higher binding energies. This could be due to the electron transfer from C_3N_4 to MnO_2.[14] As demonstrated (Supplementary Fig. 13g, 13h, 13i), significant signals of absorbed oxygen were observed (at 532.3 eV) in the O 1*s* XPS spectra, which indicated the oxygen signals could be attributed to the absorbed oxygen in these species. The absorbed oxygen could be introduced during the chemical exfoliation of bulk C_3N_4 into g-C_3N_4. In addition, the oxygen signal of metal oxides at 528-531 eV was recorded in the O 1*s* XPS spectrum of C_3N_4-MnO_2 (Supplementary Fig. 13h), while was absent in that of C_3N_4-Mn SACs (Supplementary Fig. 13i). This further confirmed the absence of MnO or MnO_2 in the C_3N_4-Mn SACs, which was in accordance with the in situ synthesis of C_3N_4-Mn SACs upon reduction of MnO_2 into Mn^{2+}.[15]

Supplementary Table 1.

Sample	Shell	CN[a]	R (Å)[b]	σ^2 (Å^2)[c]	ΔE_0 (eV)[d]	R factor
C_3N_4-Mn SACs	Mn-N	3.5	1.97	0.0019	3.47	0.008

[a] CN, coordination number; [b] R, the distance between absorber and backscatter atoms; [c] σ^2, DebyeWaller factor to account for both thermal and structural disorders; [d] ΔE_0, inner potential correction; R factor indicated the goodness of the fit. S_0^2 was fixed to 0.8. Error bounds that characterize the structural parameters obtained by EXAFS spectroscopy were estimated as CN ± 20%; σ2 ± 20%; R ± 0.04Å

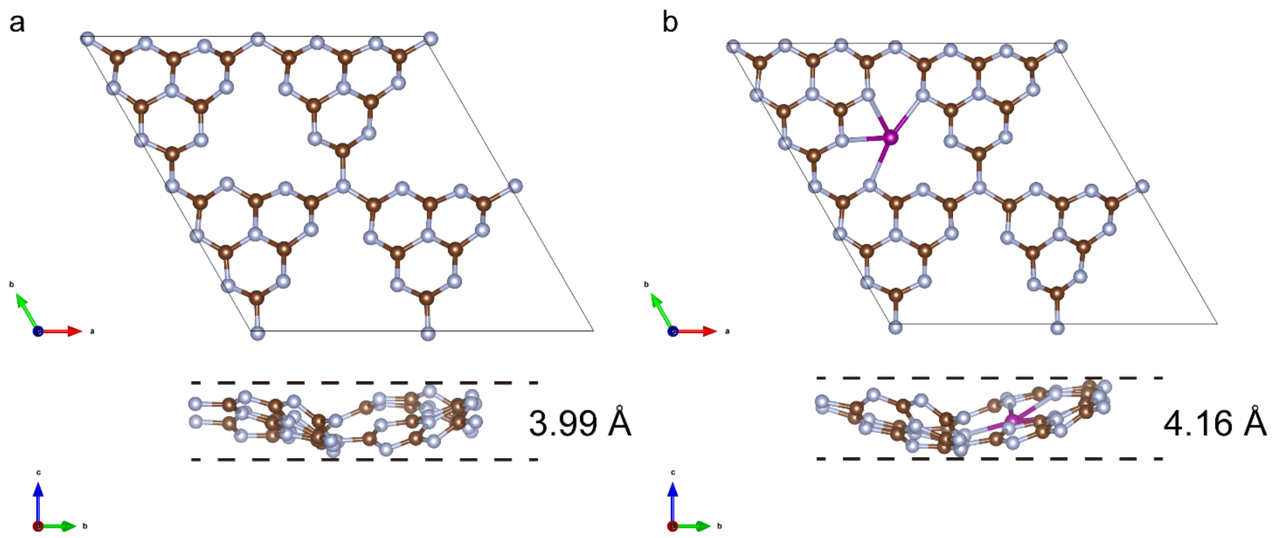

Supplementary Figure 14. Top and side views of optimized geometry of **a** C_3N_4 and **b** C_3N_4-Mn SACs. Atom colors in catalyst: C (brown), N (gray), Mn (purple).

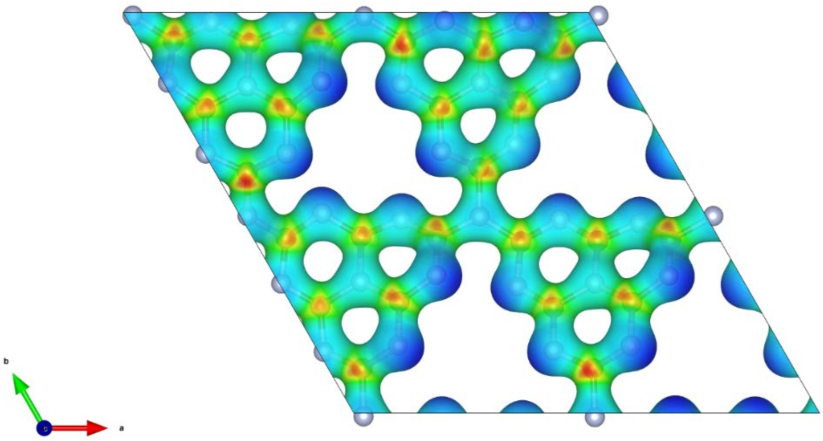

Supplementary Figure 15. Top view of the Electrostatic Potential Analysis on the molecular surface of C_3N_4. Red: positive, blue: negative. The isosurface value is taken as 0.12 e*bohr^{-3}.

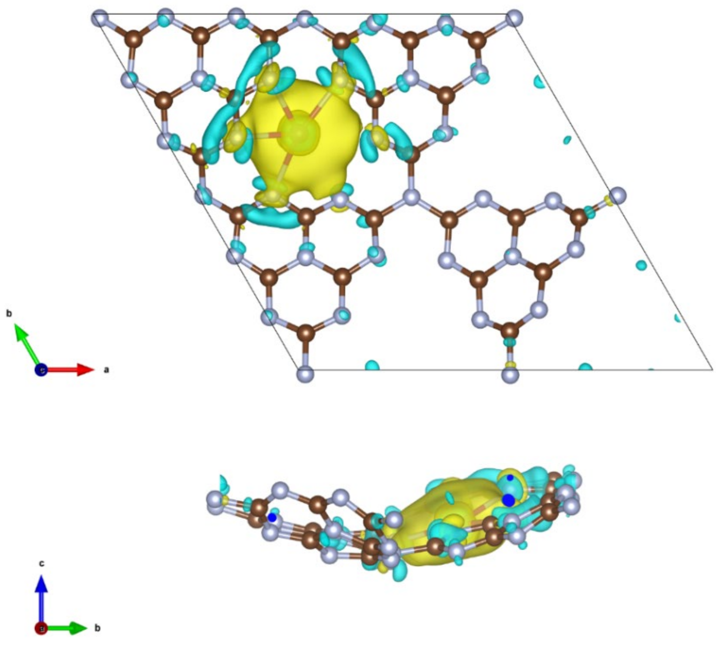

Supplementary Figure 16. Top and side views of the Charge Density Difference Analysis for the C_3N_4-Mn SACs. The yellow and the cyan areas represent charge accumulation and depletion, respectively. The isosurface value is taken as 0.0025 e*bohr^{-3}.

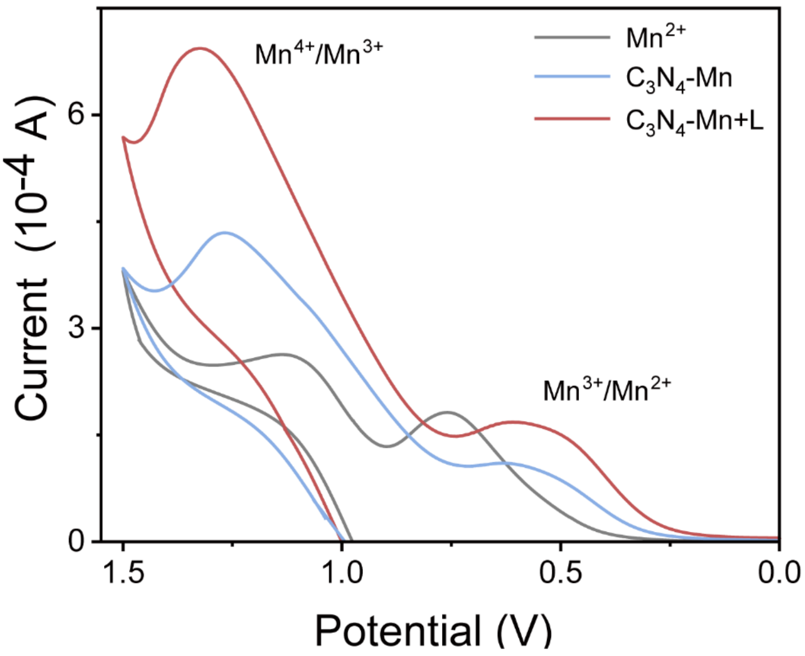

Supplementary Figure 17. CV curves of Mn^{2+} and g-C_3N_4 with or without light irradiation (660 nm, 0.4 W/cm^2). c(C_3N_4)=50 μg/mL.

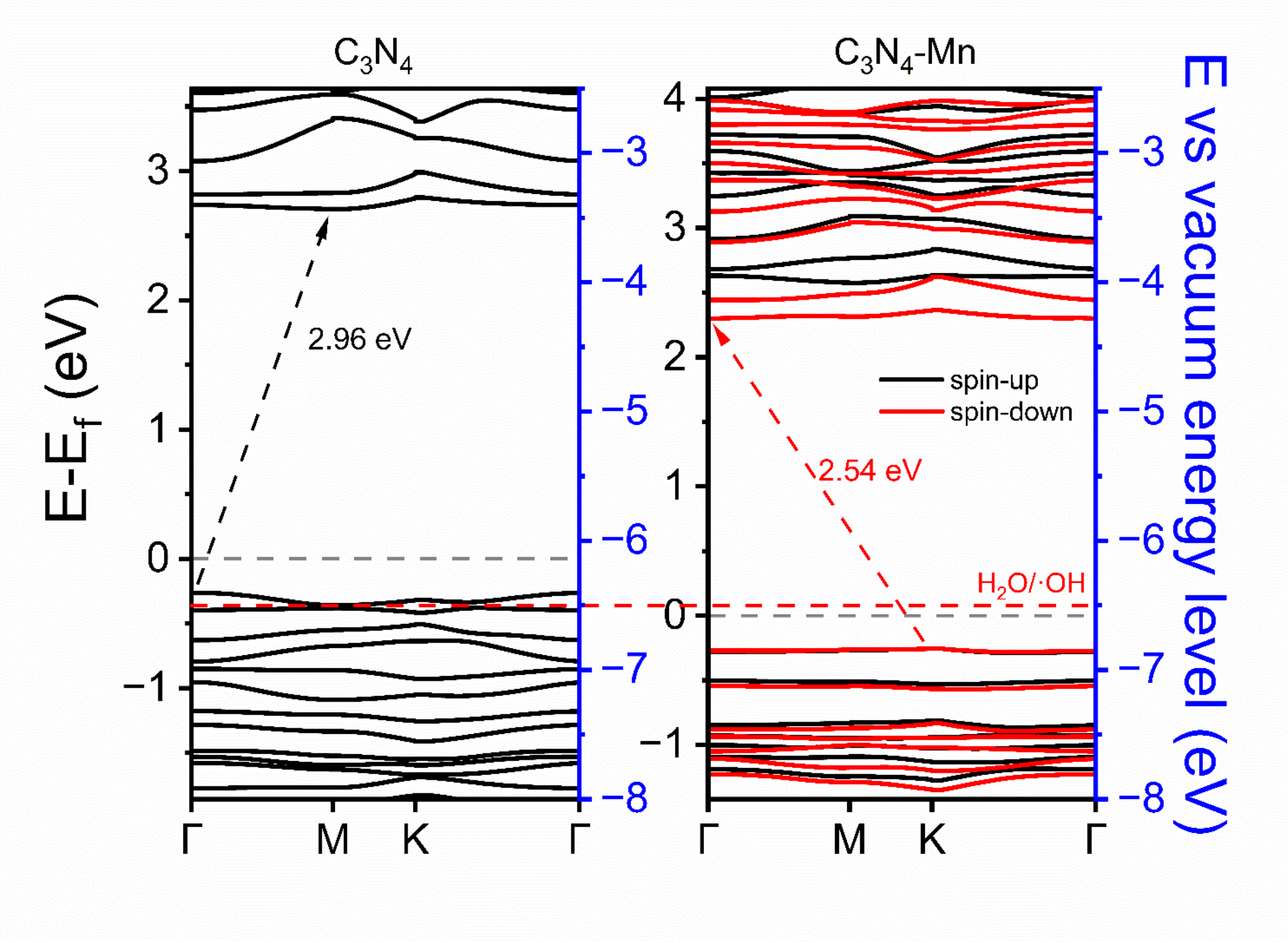

Supplementary Figure 18. PBE calculated band diagram of C_3N_4 (left) and C_3N_4-Mn (right). The gray dashed lines represent the Fermi energy level.

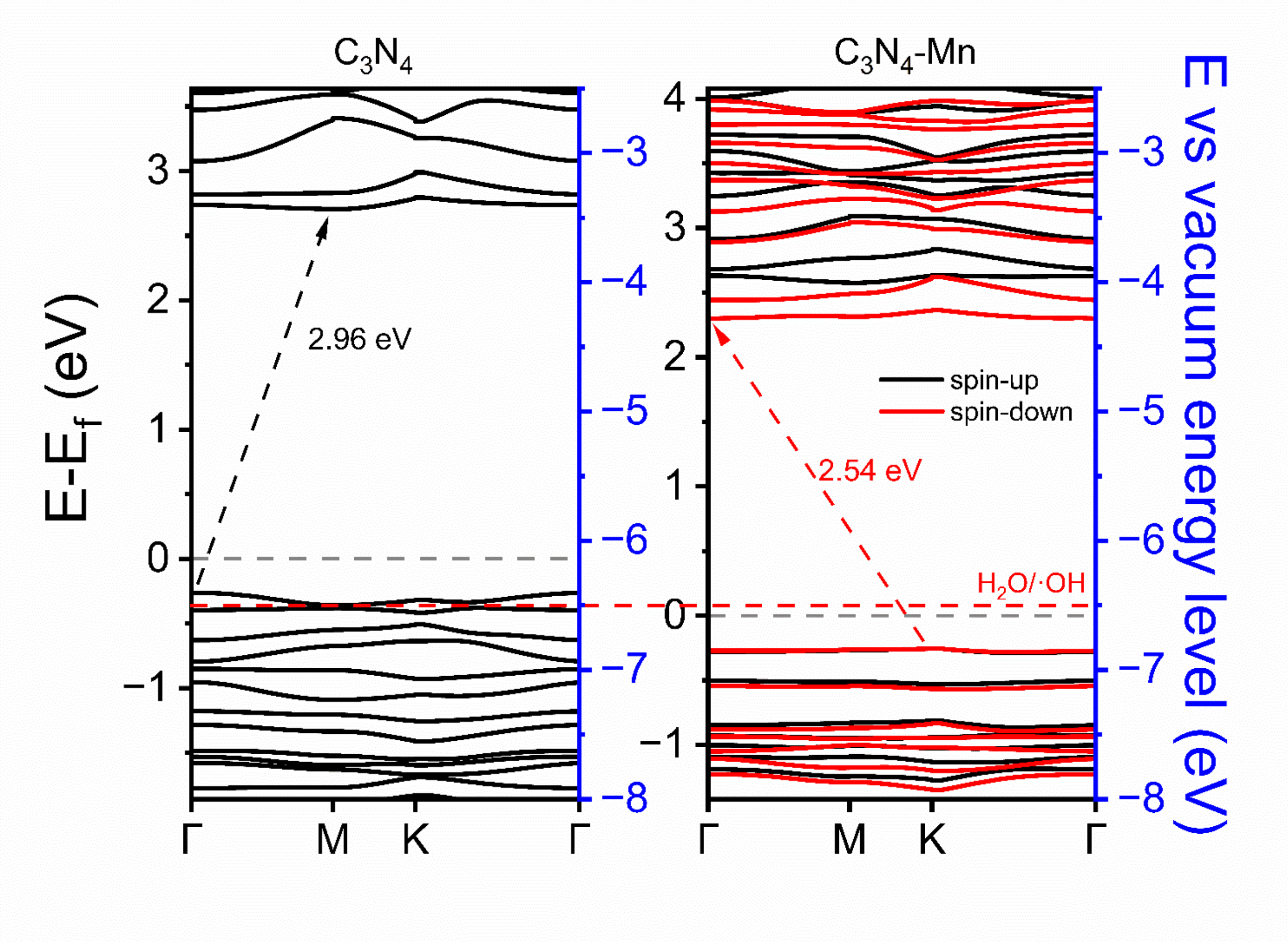

Supplementary Figure 19. HSE06 calculated band diagram of C_3N_4 (left) and C_3N_4-Mn (right). The gray dashed lines represent the Fermi energy level, and the red dashed line represents the oxidation potential of H_2O/·OH.

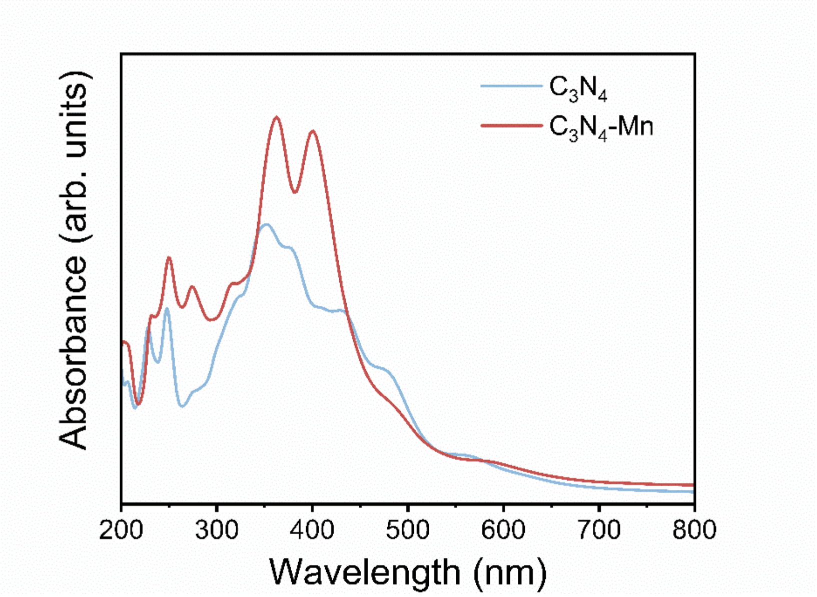

Supplementary Figure 20. The light-absorption spectra of C_3N_4 and C_3N_4-Mn based on the PBE level of theory.

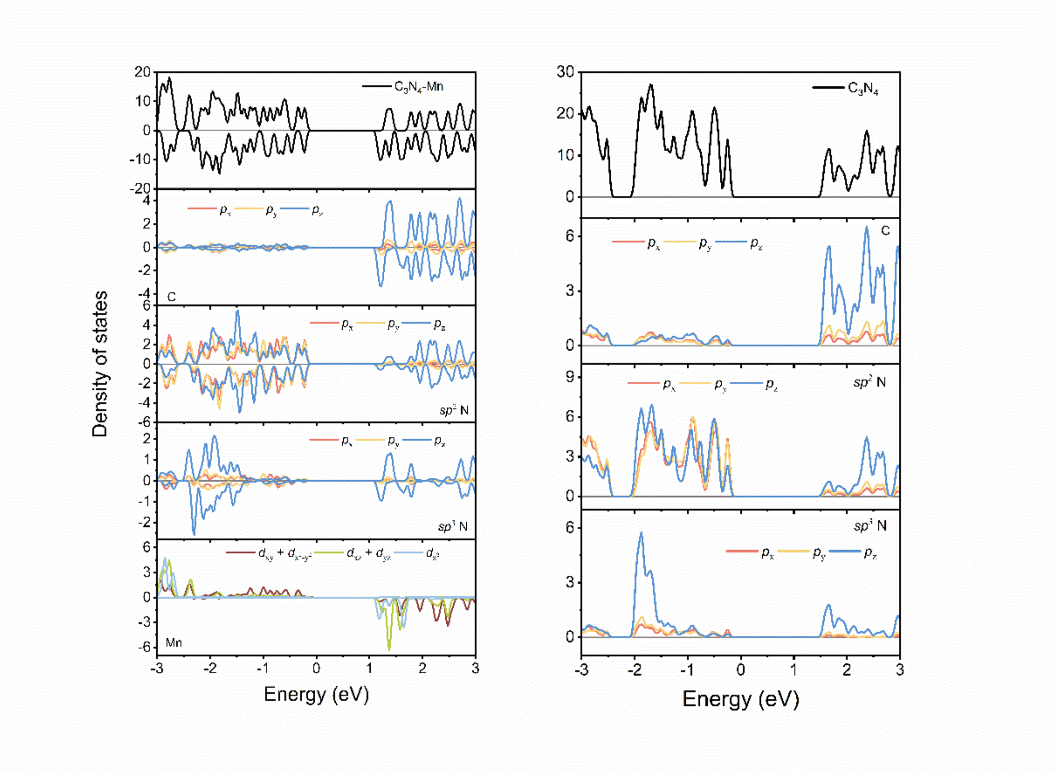

Supplementary Figure 21. PBE calculated partial density of states of C_3N_4-Mn and C_3N_4.

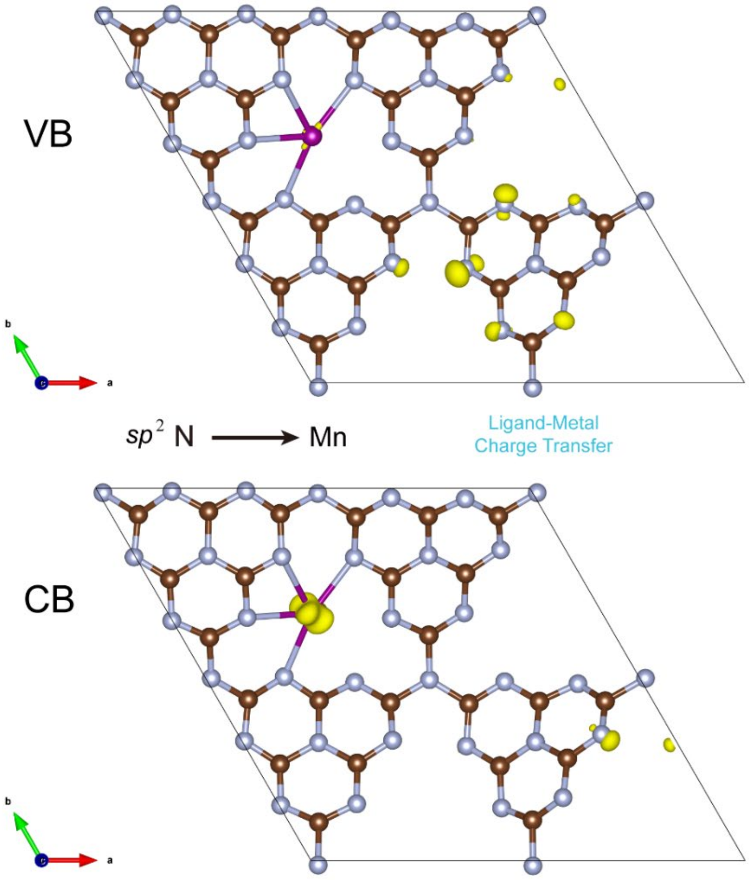

Supplementary Figure 22. PBE calculated photoexcited charge density transition from VB to CB of C_3N_4-Mn, indicating the LMCT process. The yellow bubble represents the electron population. The isosurface value is taken as 0.02 e*bohr^{-3}.

As shown in Supplementary Fig. 18 and Supplementary Fig. 19, C_3N_4 and C_3N_4-Mn are both indirect-gap materials, consistent with the formula in the Tauc Plot method (inset in Fig. 4a). As shown in Supplementary Fig. 20, the insertion of Mn single atom results in lower energy of CB for C_3N_4-Mn, which considerably reduces the excitation energy of C_3N_4-Mn SACs. This generates the red shift in the UV-Vis spectrum of C_3N_4 and C_3N_4-Mn. Furthermore, in the Excited state of C_3N_4, there is no visible separation between the electrons and holes. Because of the excellent electron transmission capacity of C_3N_4, the electrons (mainly on C) and holes (mainly on sp^2 N) generated by the irradiation may be easily recombined. While in C_3N_4-Mn SACs, a charge-separated state was generated after light excitation, which will be beneficial for further photocatalytic reactions.

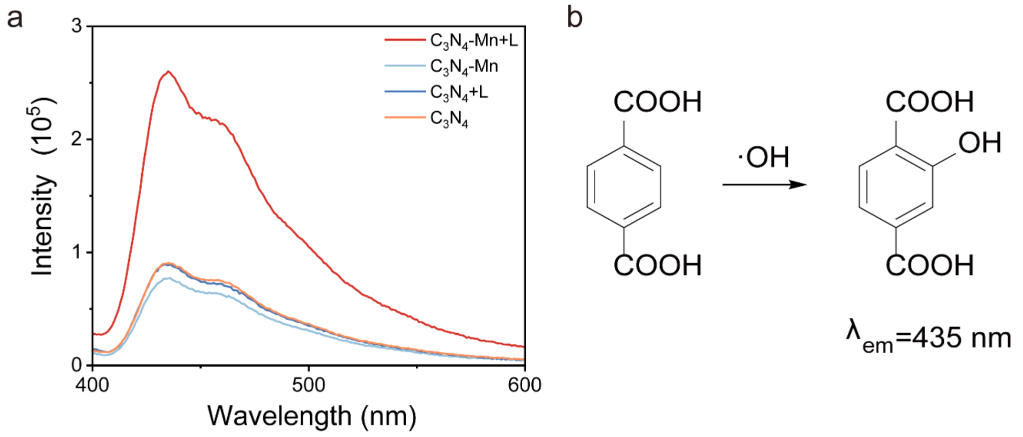

Supplementary Figure 23. a FL signals of TA from different systems with or without light irradiation (660 nm, 0.4 W/cm^2, 30 min). The experiment was repeated twice independently with similar results. **b** Schematic representation of ·OH detection by terephthalate acid (TA). [C_3N_4] = 10 μg/mL, c(TA) = 10 μM.

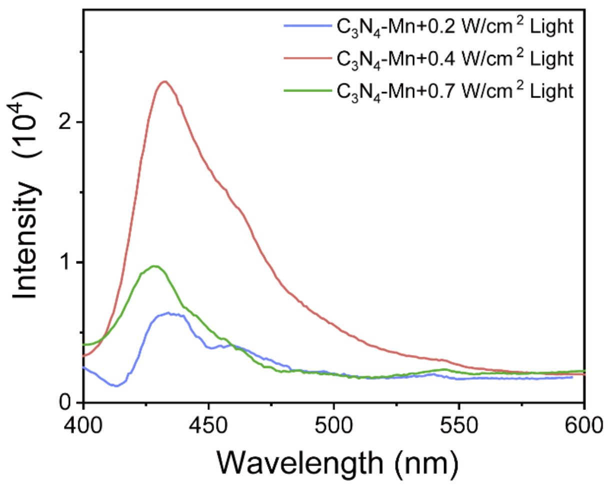

Supplementary Figure 24. The increase of the TA fluorescence intensity by C_3N_4-Mn after different irradiation (660 nm, 30 min). The experiment was repeated twice independently with similar results. c(C_3N_4) = 10 μg/mL, c(TA) = 10 μM.

As shown in Supplementary Fig. 24, the generation of ·OH by C_3N_4-Mn was promoted by increasing the light intensity (660 nm). However, when the light was excessively intense (0.7 W/cm^2), the ·OH generation exhibited a significant decline and the fluorescence signal showed a blue shift. This could be generated from the destruction of tri-s-triazine units of C_3N_4 by overexposure.

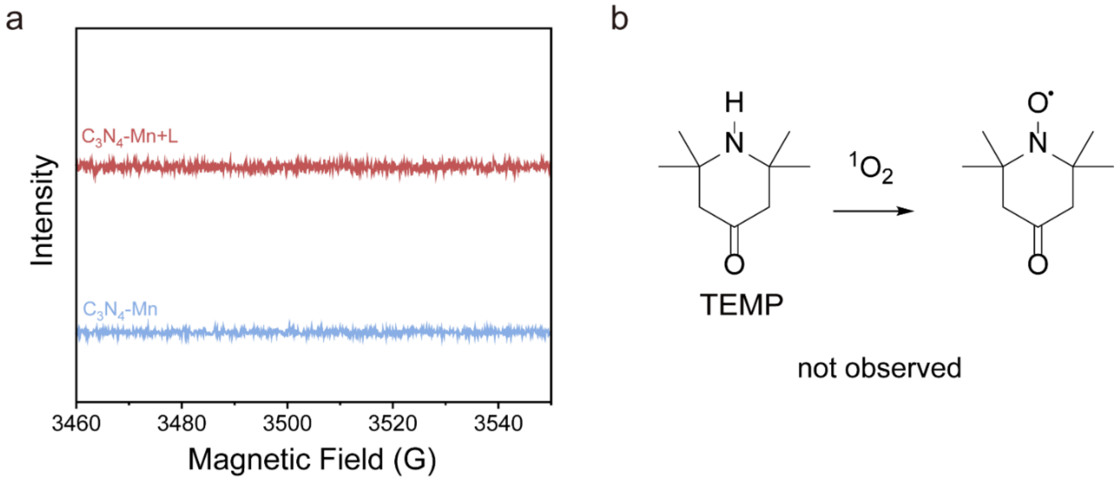

Supplementary Figure 25. a EPR spectra of C_3N_4-Mn before and after the light irradiation (660 nm, 0.4 W/cm^2) for 30 min. TEMP acted as the trapping agent. **b** Schematic representation for the detection of $^1O_2$ by TEMP.

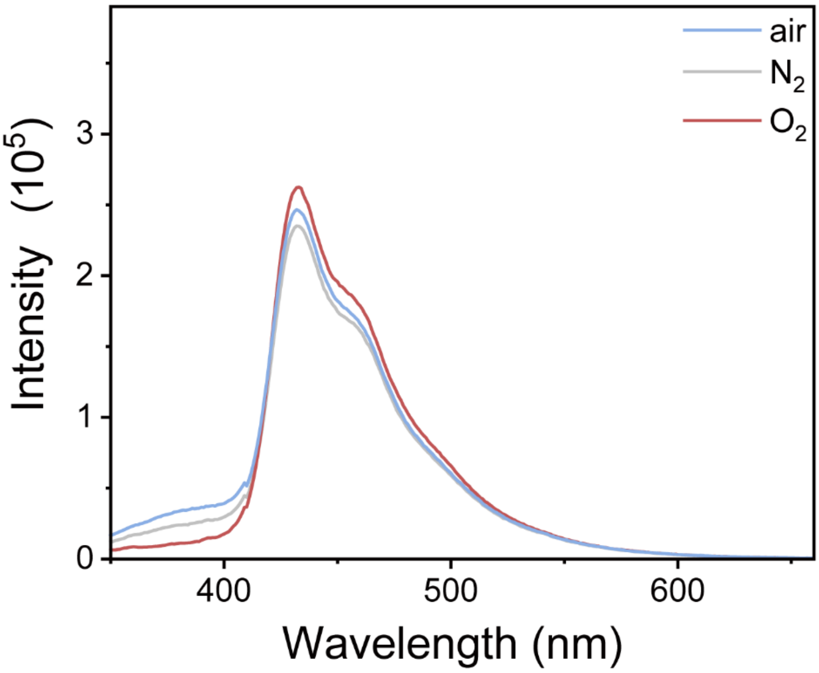

Supplementary Figure 26. Detection of ·OH by fluorescence method. The C_3N_4-Mn SACs were irradiated for 30 min (660 nm, 0.4 W/cm^2) in O_2 and N_2 environments. The experiment was repeated three times independently with similar results. c(C_3N_4) = 10 μg/mL, c(TA) = 10 μM.

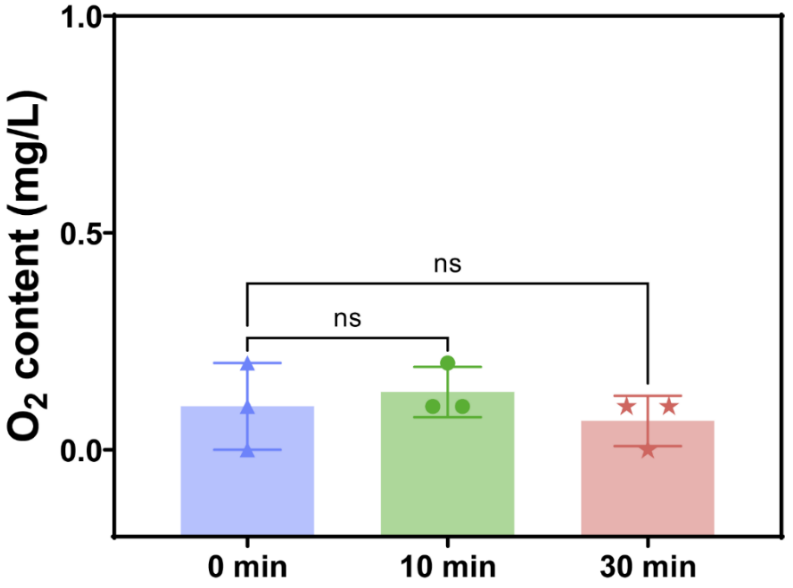

Supplementary Figure 27. Time-dependent O_2 generation by C_3N_4-Mn solution after 30 min irradiation (660 nm, 0.4 W/cm^2). c(C_3N_4) = 10 μg/mL. Data are presented as mean ± SD (n=3), ns(p>0.05): p=0.6036, 0 min vs. 10 min, ns(p>0.05): p=0.6036, 0 min vs. 30 min, one-way ANOVA multiple comparison test.

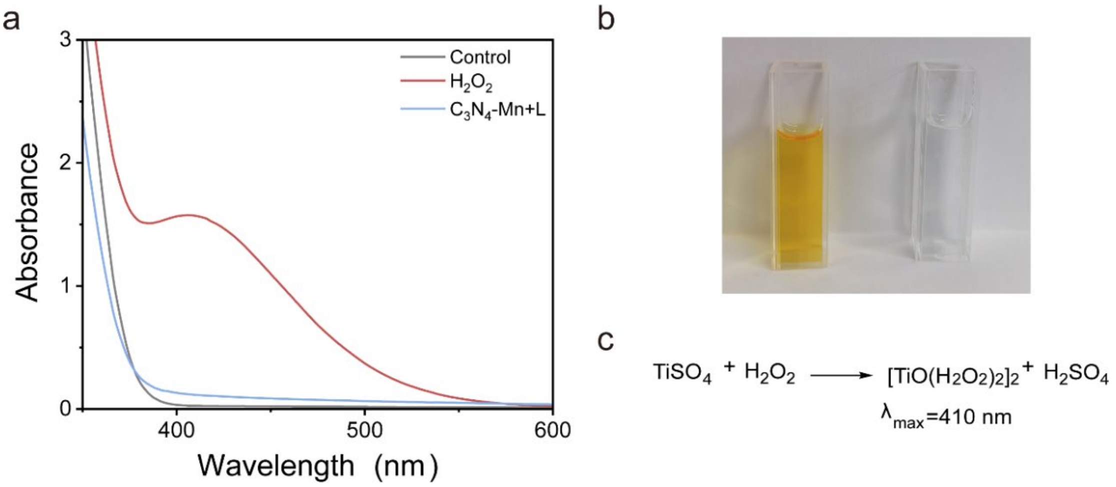

Supplementary Figure 28. a UV-vis absorption of 1% $TiSO_4$ after the addition of C_3N_4-Mn SACs and irradiated for 30 min (660 nm, 0.4 W/cm^2) or the addition of H_2O_2. The experiment was repeated three times independently with similar results. **b** The photo of the $TiSO_4$ after the addition of H_2O_2 (left) and the C_3N_4-Mn after irradiating for 30 min (660 nm, 0.4 W/cm^2) (right). **c** Schematic representation of H_2O_2 detection by $TiSO_4$. c(H_2O_2) = 1mM, c(C_3N_4) = 10 μg/mL.

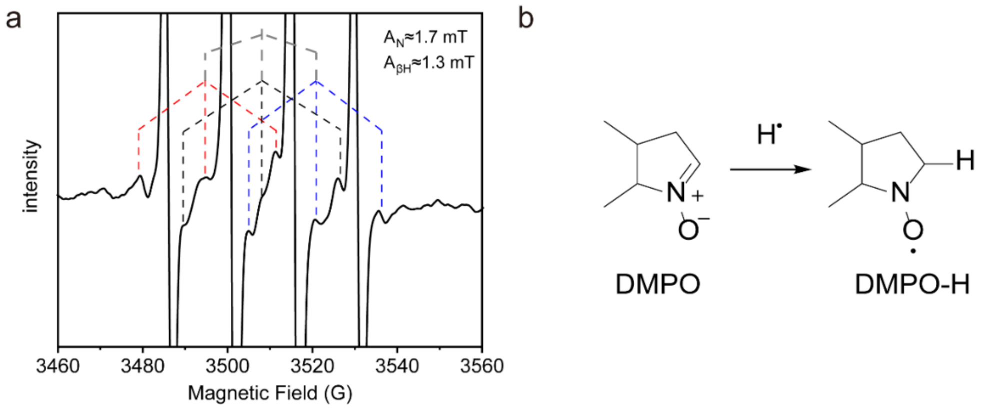

Supplementary Figure 29. a EPR signal of the mixture containing DMPO and C_3N_4-Mn under 660 nm light irradiation (0.4 W/cm^2) for 30 min. **b** Schematic representation on detection of ·H by 5,5-dimethyl-1-pyrroline N-oxide (DMPO). c(C_3N_4) = 10 μg/mL, c(DMPO) = 100 mM.

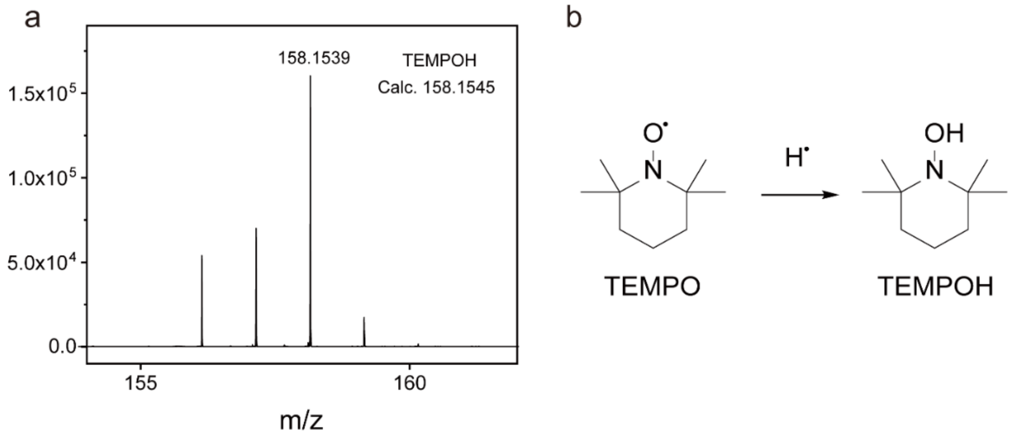

Supplementary Figure 30. a HRMS signal of the mixture containing TEMPO and C_3N_4-Mn under 660 nm light irradiation (0.4 W/cm^2) for 30 min. **b** Schematic representation of ·H detection by 2,2,6,6-Tetramethylpiperidine 1-oxyl (TEMPO). c(C_3N_4) = 10 μg/mL, c(TEMPO) = 10 mM.

As shown in Supplementary Fig. 28, the DMPO-H adduct was recorded after irradiation of C_3N_4-Mn solutions. This indicated the generation of ·H according to the characteristic hyperfine splitting constants of the nine-line signal (A_N ≈ 1.7 G, $A_{\beta H}$ ≈ 1.3 G). The generation of ·H was also demonstrated by observing TEMPOH adduct (m/z 158.1545) in the HRMS spectrum (Supplementary Fig. 29). Consequently, along with the generation of ·OH, ·H was also produced simultaneously.

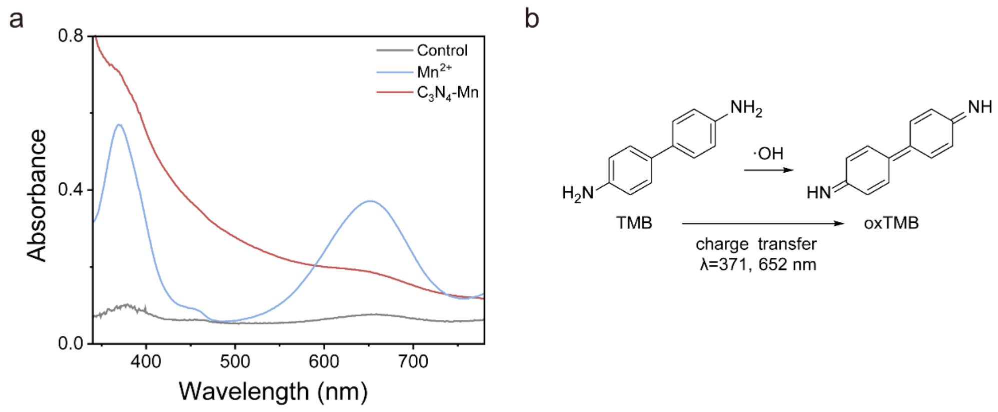

Supplementary Figure 31. a UV-vis absorption of TMB in H_2O_2 with Mn^{2+} or C_3N_4-Mn SACs presented. The experiment was repeated three times independently with similar results. **b** Schematic representation of ·OH detection by 3,3',5,5'-Tetramethylbenzidine (TMB), the increased absorption of TMB at 371 nm and 652 nm (by the charge transfer complex from TMB to oxTMB) indicated the generation of ·OH. c(C_3N_4) = 10 μg/mL, c(TMB) = 10 μM.

Without the irradiation, no ·OH could be generated from the Fenton-like reaction in H_2O_2. This could be attributed to the decreased electrostatic attractions between anionic H_2O_2 and metal sites with high electron density (Supplementary Fig. 30).

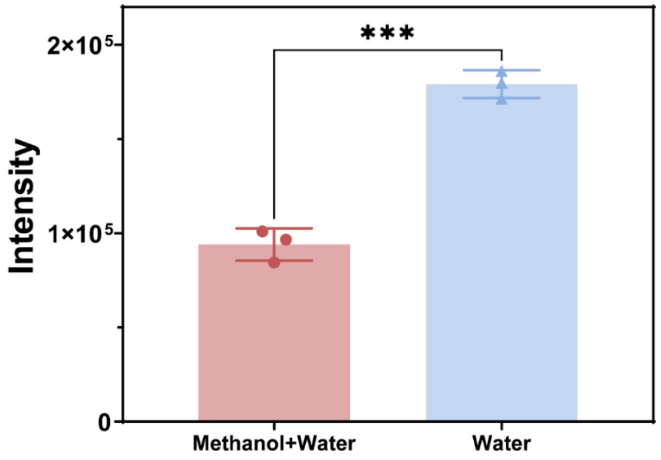

Supplementary Figure 32. The generation of ·OH by C_3N_4-Mn after 30 min irradiation (660 nm, 0.4 W/cm^2) was evaluated by FL intensities of TA with and without methanol as the hole scavenger. Data are presented as mean ± SD (n=3). c(C_3N_4-Mn) = 10 μg/mL, c(TA) = 10 μM. ***($p<0.0021$): p=0.0002, Methanol+Water vs. Water, two-tailed t comparison test.

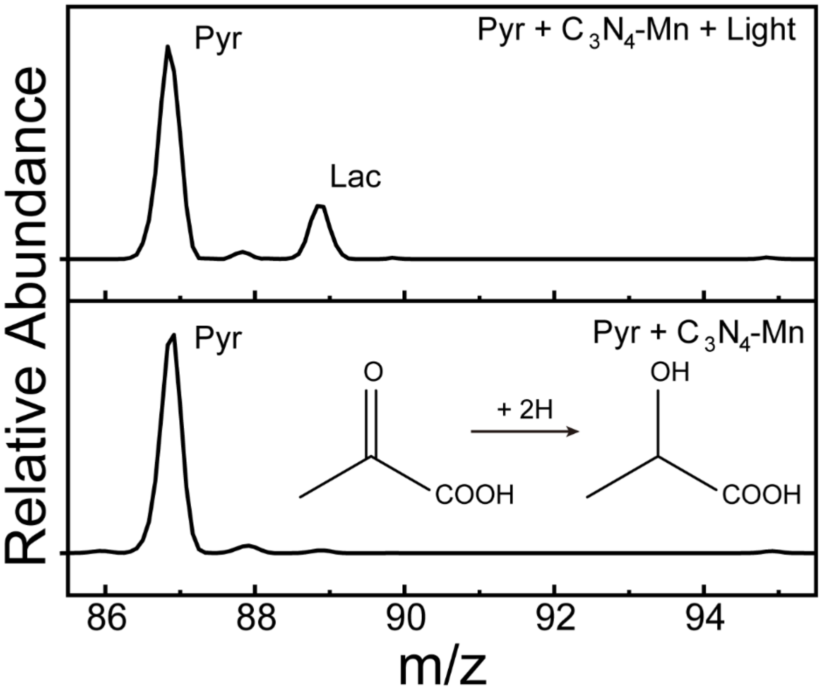

Supplementary Figure 33. The MS spectra of Pyr and C_3N_4-Mn SACs before and after light irradiation (660 nm, 0.4 W/cm^2) for 30 min. c(C_3N_4-Mn) = 10 μg/mL, c(Pyr) = 1 mM.

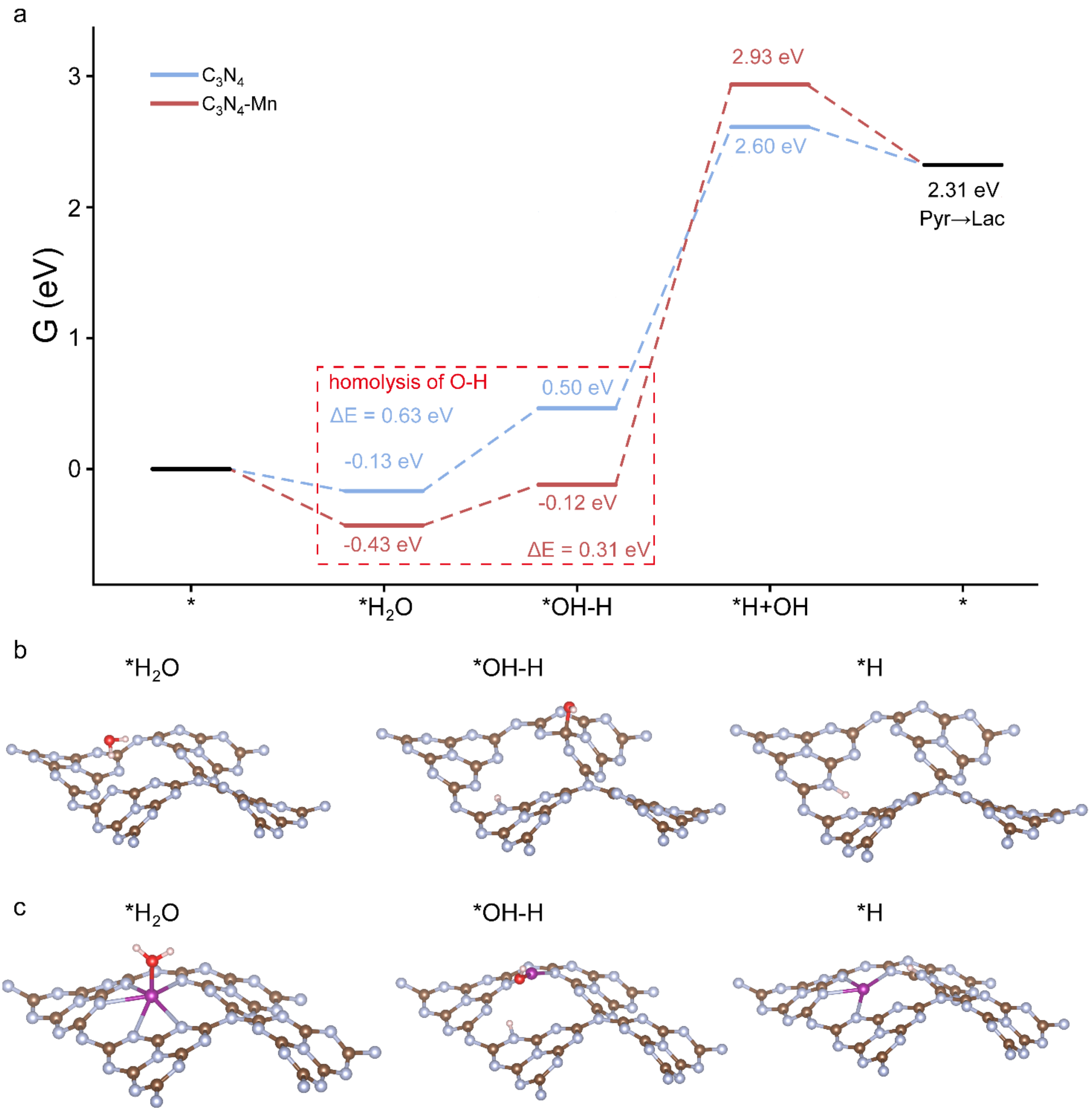

Supplementary Figure 34. a Energy profiles of photocatalytic water splitting for ·OH generation on the surface of C_3N_4 and C_3N_4-Mn, along with the comparison of the reduction of Pyr. Illustrations of reaction pathways of the photocatalytic water splitting over **b** C_3N_4 and **c** C_3N_4-Mn. Atom colors in catalyst: C (brown), N (gray), Mn (purple), O (red), H (white).

The detailed reaction path of the water splitting on the surface of C_3N_4 and C_3N_4-Mn was

determined. As shown in Supplementary Fig. 33, C_3N_4-Mn was easier to adsorb water molecules (with a free energy change of -0.43 eV) than C_3N_4 (with a free energy change of -0.13 eV). This could be generated from the positive charge on Mn, which slightly enhanced the absorption of C_3N_4-Mn for polar small molecules (water). Clearly, C_3N_4-Mn had much lower Gibbs free energy for the ·OH generation. In detail, at a free energy change of 0.31 eV (-0.43 to -0.12 eV), the homolysis of the O-H bond over C_3N_4-Mn is much easier than over C_3N_4 (0.63 eV, -0.13 to 0.50 eV). It is the critical step in the generation of ·OH and the significant difference indicates that the introduction of Mn can significantly enhance the catalytic capability of ·OH generation. Correspondingly, C_3N_4-H-Mn demonstrates an excellent capability to reduce Pyr, which is confirmed by the lower free energy change (-0.62 eV, 2.93 to 2.31 eV). Therefore, the water splitting catalyzed by C_3N_4-Mn SACs can generate ·OH effectively, and subsequently Pyr can be reduced by C_3N_4-H-Mn. Consequently, along with maintaining the photocatalytic cycles, cellular respiration of cancer cells can be blocked upon consuming a significant energy substance of Pyr.

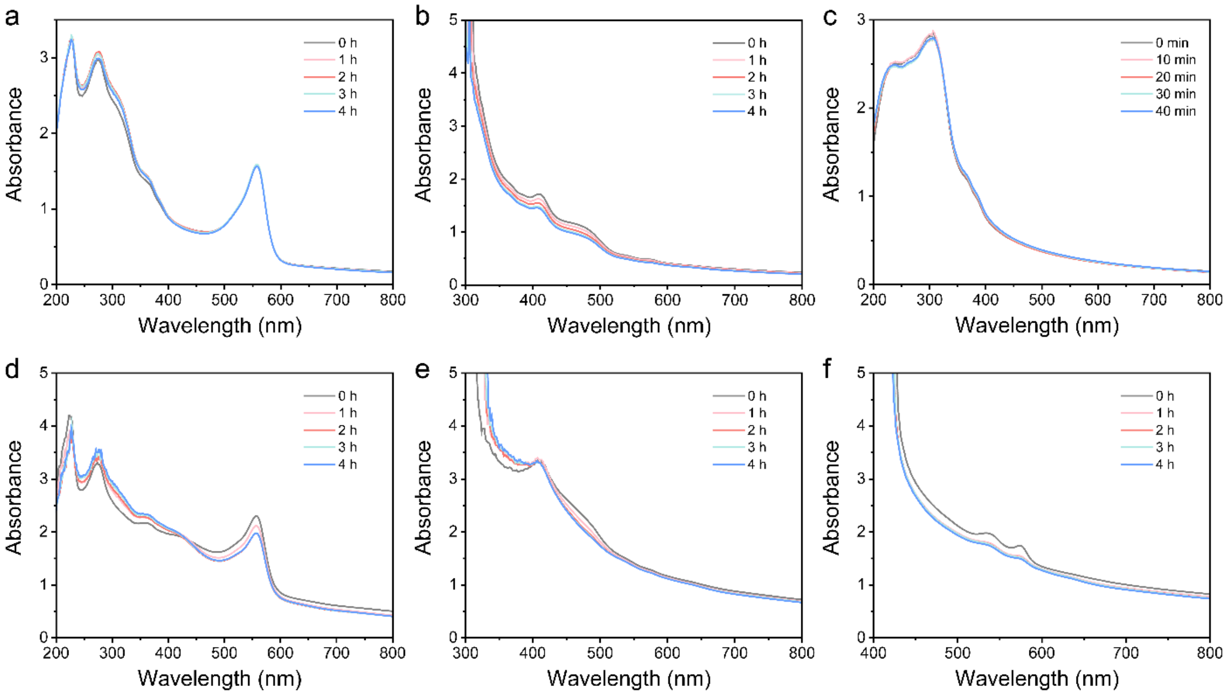

Supplementary Figure 35. UV-vis absorption of C_3N_4-Mn in **a** DMEM Culture medium and **b** FBS from 0 to 4 h. **c** UV-vis absorption of C_3N_4-Mn in PBS after light irradiation (660 nm, 0.4 W/cm^2) for 0 to 40 min. UV-vis absorption of C_3N_4-MnO_2 in **d** DMEM culture medium, **e** FBS, and **f** mouse plasma from 0 to 4 h. The experiment was repeated twice independently with similar results.

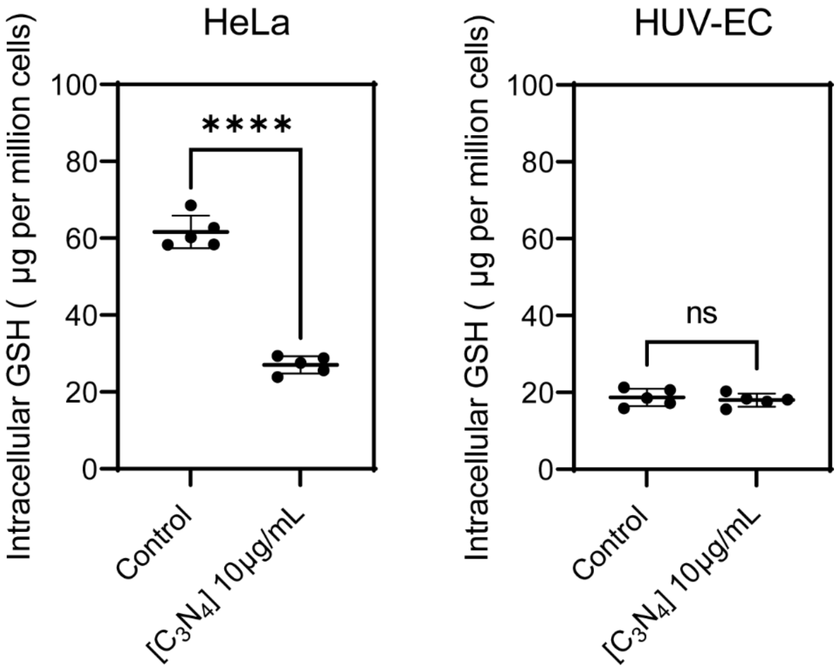

Supplementary Figure 36. Intracellular GSH concentration levels in HeLa cells and HUV-EC cells before and after 24 h of the incubation with C_3N_4-MnO_2. [C_3N_4] = 10 μg/mL. Data are presented as mean ± SD (n=5 biologically independent samples). ****($p<0.0001$): HeLa, ns($p>0.05$): p=0.5944, HUV-EC, two-tailed t comparison test.

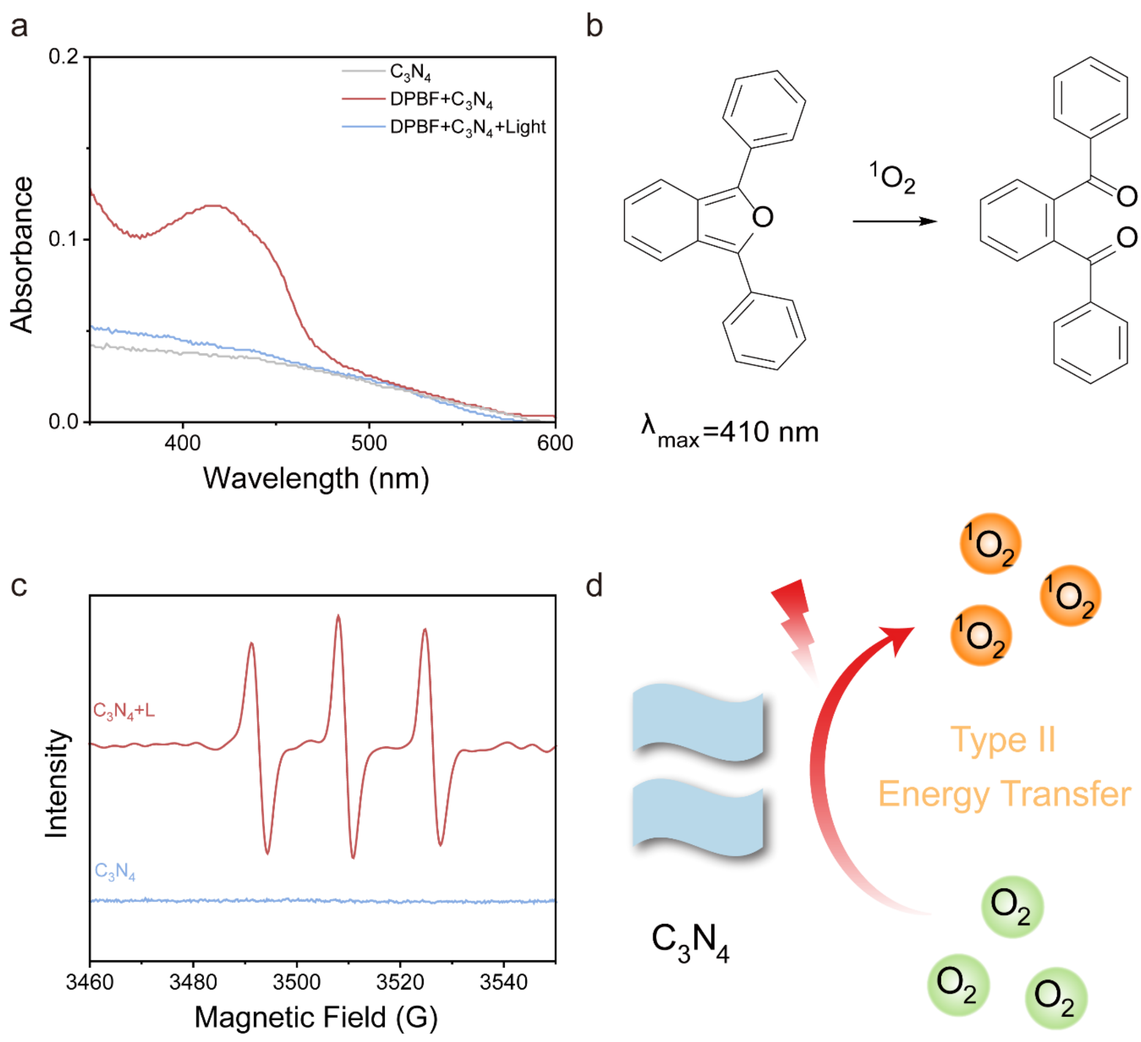

Supplementary Figure 37. **a** UV-vis spectra of DPBF in C_3N_4 after irradiation for 30 min (635 nm, 0.4 W/cm^2). The experiment was repeated twice independently with similar results. **b** Schematic representation of $^1O_2$ detection by DPBF. **c** EPR spectra of C_3N_4 before and after the light irradiation (635 nm, 0.4 W/cm^2) for 30 min. TEMP acted as the trapping agent. **d** The schematic illustration of the generation of $^1O_2$ by the C_3N_4 under light irradiation. c(C_3N_4) = 100 μg/mL, c(DPBF) = 10 μM.

As shown in Supplementary Fig. 37, C_3N_4 can convert O_2 to $^1O_2$ under 635 nm light irradiation, which facilitated the Type-II PDT. The specific reagent of DPBF was selected for detecting singlet oxygen, upon the absorption of the singlet oxygen oxidized produced at 410 nm (Supplementary Figure 3b). As a result (Supplementary Figure 37a), the significantly increased absorption at 410 nm was recorded after 30 min of the light irradiation on C_3N_4, confirming the generation of singlet oxygen. Besides, the EPR analysis of singlet oxygen was carried out with TEMP as the trapping agent. As shown in Supplementary Figure 37c, the significant triple peak of TEMPO· was observed in the EPR spectrum after light irradiation for 30 min, verifying the presence of singlet oxygen. These results are in accordance with the light irradiated producing of ROS by C_3N_4 under normoxia relative to the failure of ROS generation under hypoxia.

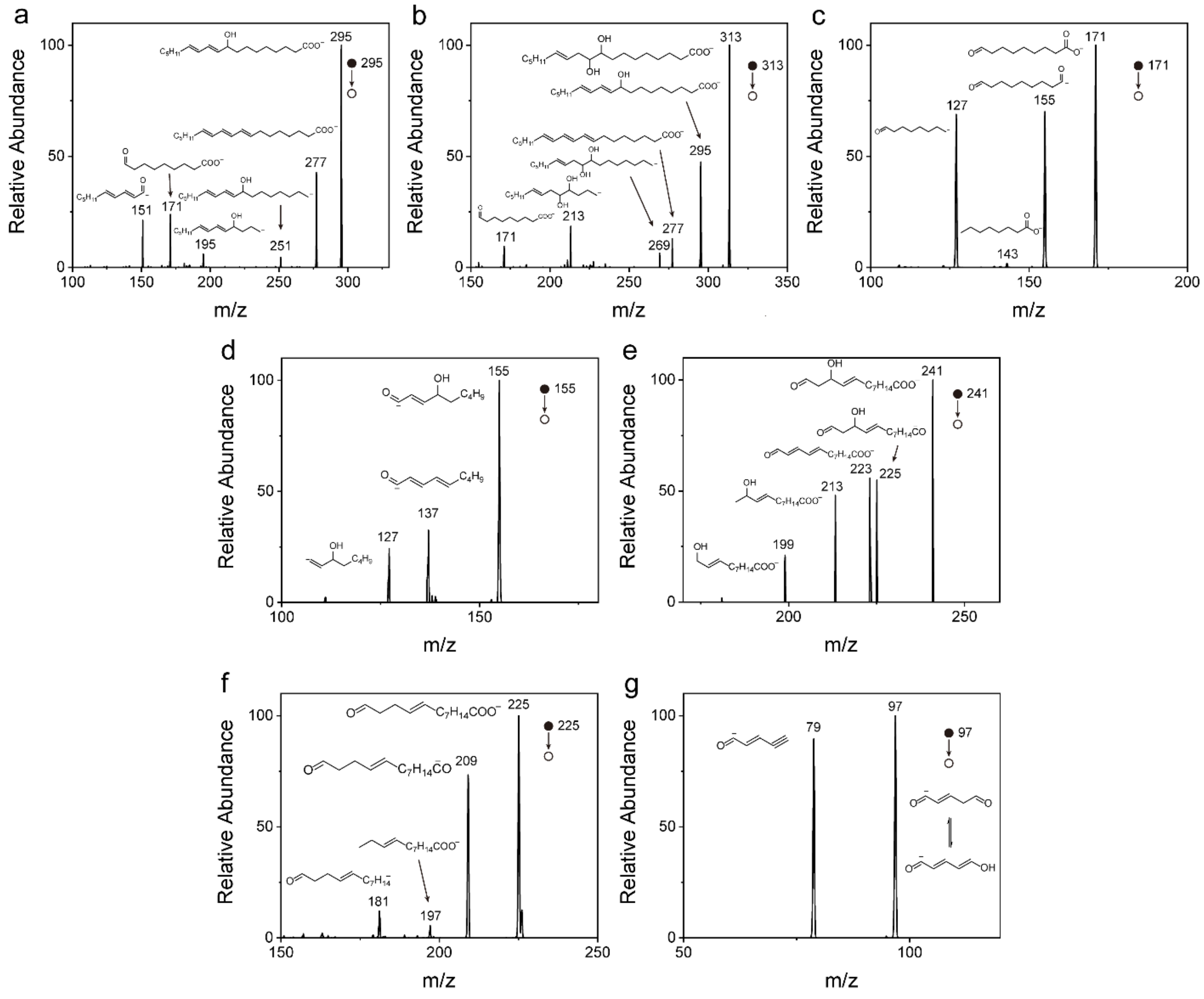

Supplementary Figure 38. CID products of ions at **a** m/z 295, **b** m/z 313, **c** m/z 171, **d** m/z 155 (4-HNE), **e** m/z 241, **f** m/z 225, and **g** m/z 97, showing that the structure of intermediates during the LPO process.

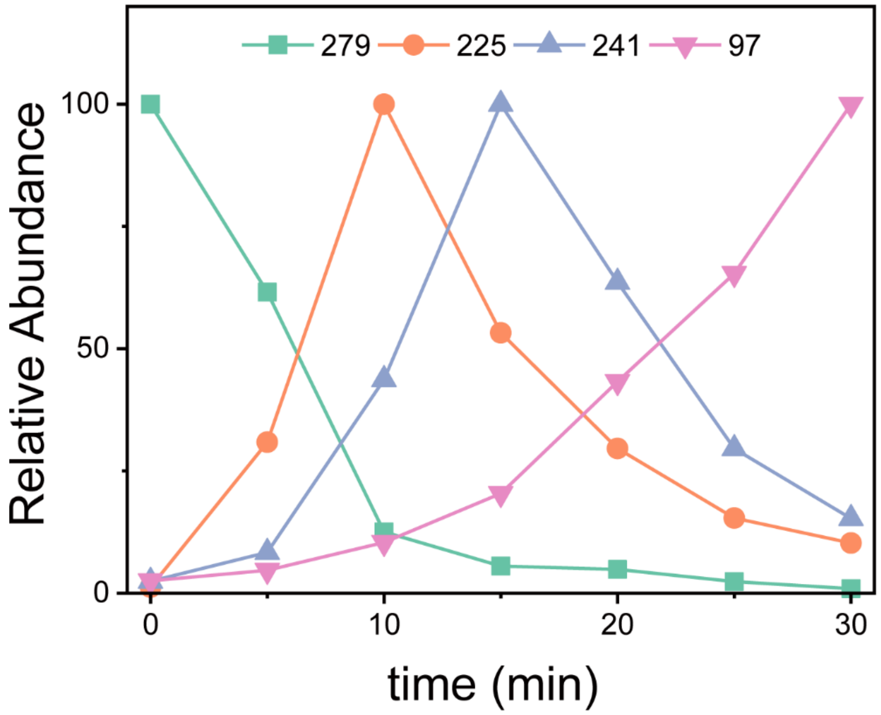

Supplementary Figure 39. Time-dependent MS signal of ions at m/z 279, 225, 241, and 97 after the addition of C_3N_4-Mn and LA. Irradiation: 660 nm, 0.4 W/cm^2, 30 min. c(C_3N_4-Mn) = 10 μg/mL, c(LA) = 100 μM.

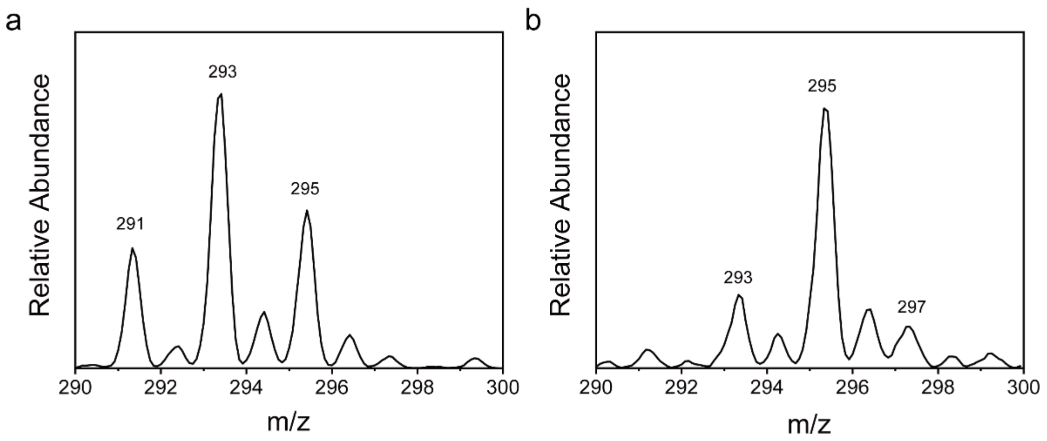

Supplementary Figure 40. MS spectra of LA in H_2O and $H_2{}^{18}O$ with C_3N_4-Mn and LA added, which were recorded after irradiation for 30 min (660 nm, 0.4 W/cm^2). The experiment was repeated twice independently with similar results. c(C_3N_4-Mn) = 10 μg/mL, c(LA) = 100 μM.

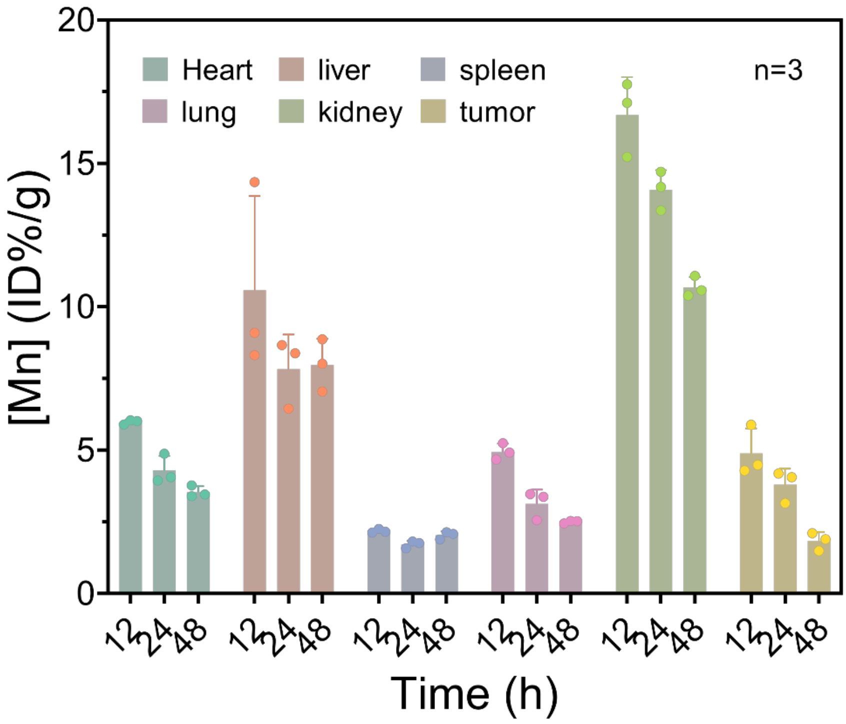

Supplementary Figure 41. The biodistribution of C_3N_4-MnO_2 by measuring Mn concentrations over 12, 24, and 48 h after the intravenous injection. Data are presented as mean ± SD (n=3 biologically independent animals).

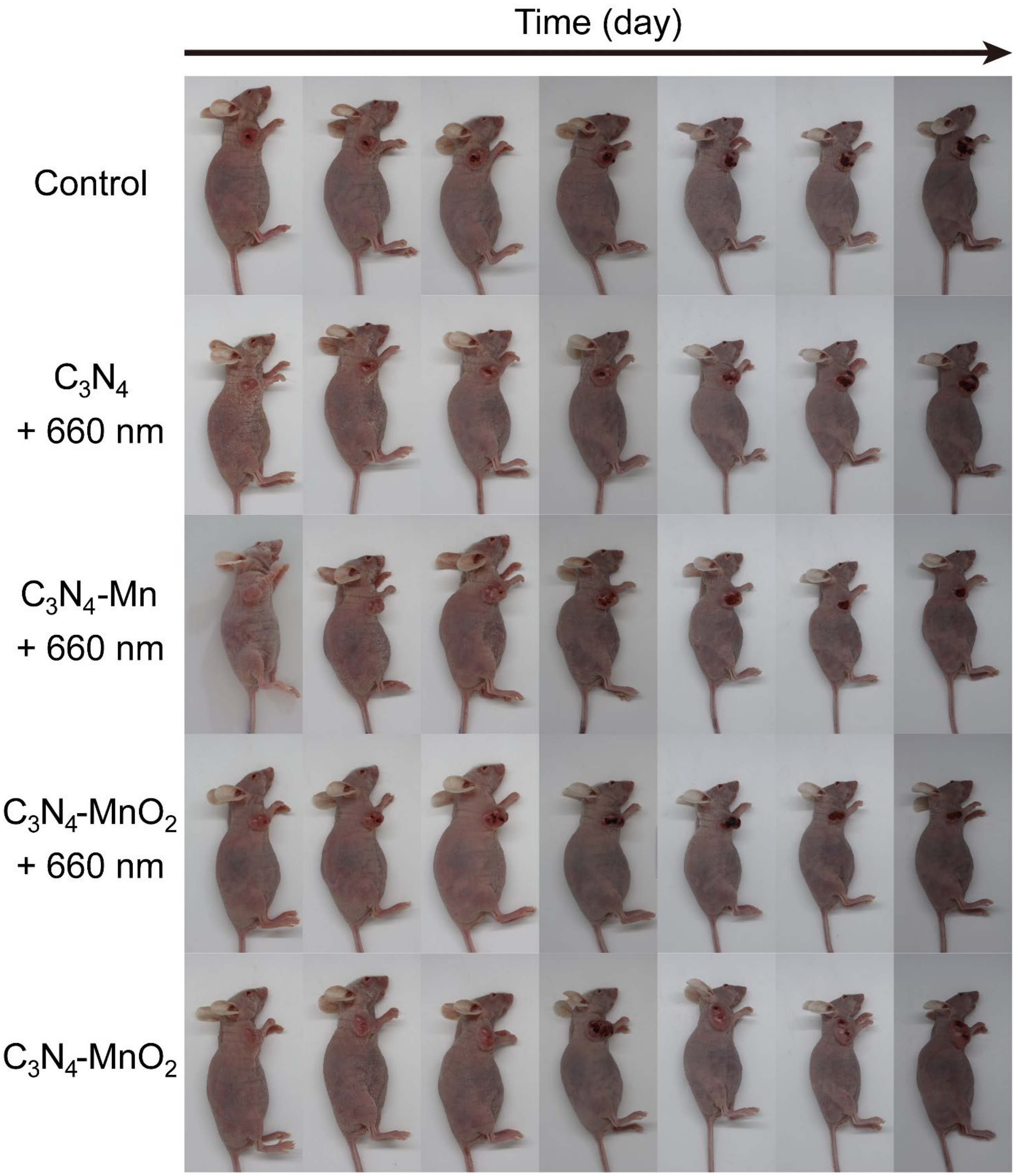

Supplementary Figure 42. Representative images of mice after different treatments. The experiment was repeated three times independently with similar results.

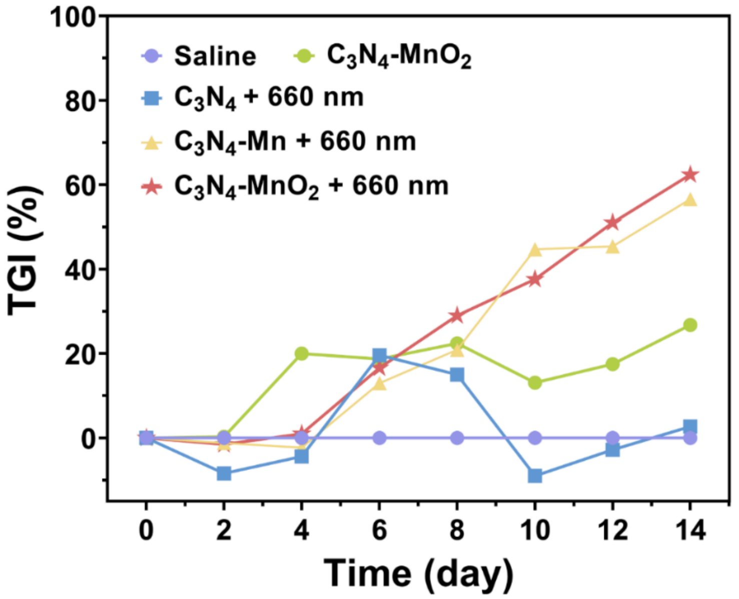

Supplementary Figure 43. The TGI% changes of HeLa tumor-bearing mice after different treatments in 14 days.

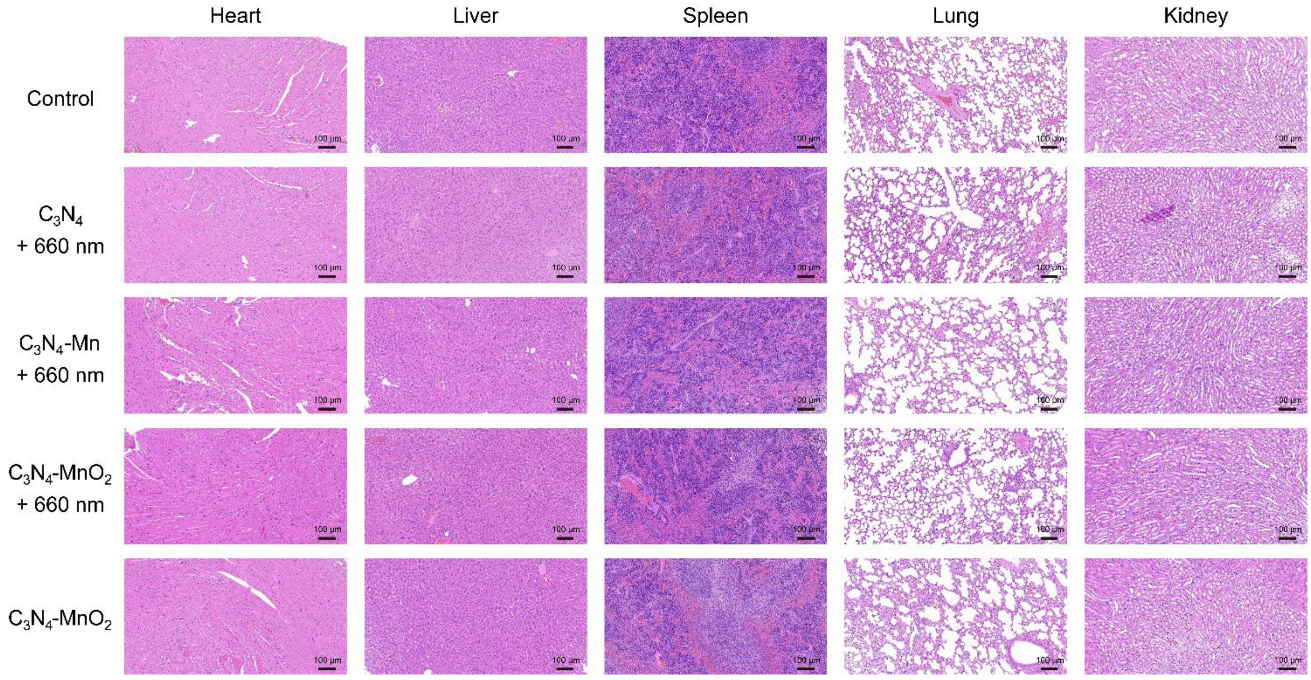

Supplementary Figure 44. Representative H&E-stained images of organs obtained from mice in each group at 14 days. The experiment was repeated three times independently with similar results.

Supplementary References

1. Wang, Y., Sun, M., Qiao, J., Ouyang, J. & Na, N. FAD roles in glucose catalytic oxidation studied by multiphase flow of extractive electrospray ionization (MF-EESI) mass spectrometry. *Chem. Sci.* **9**, 594-599 (2018).
2. Funke, H., Chukalina, M. & Rossberg, A. Wavelet analysis of extended x-ray absorption fine structure data. *Phys. Scr.* **T115**, 232-234 (2005).
3. Kresse, G. & Furthmüller, J. Efficiency of ab-initio total energy calculations for metals and semiconductors using a plane-wave basis set. *Jcomput. Mater. Sci.* **6**, 15-50 (1996).
4. Kresse, G. & Joubert, D. From uultrasoft pseudopotentials to the projector augmented-wave method. *Phys. Rev. B* **59**, 1758 (1999).
5. Perdew, J. P., Burke, K. & Ernzerhof, M. Generalized Gradient Approximation Made Simple. *Phys. Rev. Lett.* **77**, 3865 (1996).
6. Heyd, J., Scuseria, G. E. & Ernzerhof, M. Erratum: “Hybrid functionals based on a screened Coulomb potential”. *J. Chem. Phys.* **124**, 219906 (2006).
7. Mathew, K., Kolluru, V. S. C., Mula, S., Steinmann, S. N., & Hennig, R. G. Implicit self-consistent electrolyte model in plane-wave density-functional theory. *J. Chem. Phys.* **151**, 234101 (2019).
8. Zhang, X.-L. et al. Turn-On Fluorescence Sensor for Intracellular Imaging of Glutathione Using g-C3N4 Nanosheet-MnO2 Sandwich Nanocomposite. *Anal. Chem.* **86**, 3426-3434 (2014).
9. Sun, J., Yin, Y., Li, W., Jin, O. & Na, N. CHEMICAL REACTION MONITORING BY AMBIENT MASS SPECTROMETRY. *Mass Spectrom. Rev.* **41**, 70-99 (2022).
10. Zhu G. et al. Encapsulate α-MnO2 nanofiber within graphene layer to tune surface electronic structure for efficient ozone decomposition. *Nat. Commun.* **12**, 4152 (2021).
11. Mo, Z. et al. Synthesis of g-C3N4 at different temperatures for superior visible/UV photocatalytic performance and photoelectrochemical sensing of MB solution. *RSC Adv.* **5**, 101552-101562 (2015).
12. Jin, Y. et al. Joint Charge Storage for High-Rate Aqueous Zinc-Manganese Dioxide Batteries. *Adv. Mater.* **31** (2019).
13. Jiang, M., Yan, D., Lv, X., Gao, Y. & Jia, H. Recognition of water-dissociation effect toward lattice oxygen activation on single-atom Co catalyst in toluene oxidation. *Appl. Catal. B* **319** (2022).
14. Shi, Y. et al. Rooting MnO2 into protonated g-C3N4 by intermolecular hydrogen bonding for endurable supercapacitance. *Nano Energy* **77** (2020).
15. Li, Y. et al. Implementing Metal-to-Ligand Charge Transfer in Organic Semiconductor for Improces Visible-Near-Infrared Photocatalysis. *Adv. Mater.* **28**, 6959-6965 (2016).
