# Supplementary material for: Tumor-activated in situ synthesis of single-atom catalysts for O2-independent photodynamic therapy based on water-splitting
Source: Nat Commun. 2024 Apr 6;15:2954. doi: 10.1038/s41467-024-46987-1 (PMC11258260; doi:10.1038/s41467-024-46987-1)
Supplement: Supplementary file 2 — Reporting Summary [file 41467_2024_46987_MOESM2_ESM.pdf]

nature portfolio

Corresponding author(s): Na, Na

Last updated by author(s): Mar 7, 2024

Reporting Summary

Nature Portfolio wishes to improve the reproducibility of the work that we publish. This form provides structure for consistency and transparency in reporting. For further information on Nature Portfolio policies, see our Editorial Policies and the Editorial Policy Checklist.

Statistics

For all statistical analyses, confirm that the following items are present in the figure legend, table legend, main text, or Methods section.

n/a	Confirmed	
☐	☒	The exact sample size (n) for each experimental group/condition, given as a discrete number and unit of measurement
☐	☒	A statement on whether measurements were taken from distinct samples or whether the same sample was measured repeatedly
☐	☒	The statistical test(s) used AND whether they are one- or two-sided<br>*Only common tests should be described solely by name; describe more complex techniques in the Methods section.*
☐	☒	A description of all covariates tested
☒	☐	A description of any assumptions or corrections, such as tests of normality and adjustment for multiple comparisons
☐	☒	A full description of the statistical parameters including central tendency (e.g. means) or other basic estimates (e.g. regression coefficient) AND variation (e.g. standard deviation) or associated estimates of uncertainty (e.g. confidence intervals)
☐	☒	For null hypothesis testing, the test statistic (e.g. F, t, r) with confidence intervals, effect sizes, degrees of freedom and P value noted<br>*Give P values as exact values whenever suitable.*
☒	☐	For Bayesian analysis, information on the choice of priors and Markov chain Monte Carlo settings
☒	☐	For hierarchical and complex designs, identification of the appropriate level for tests and full reporting of outcomes
☒	☐	Estimates of effect sizes (e.g. Cohen's d, Pearson's r), indicating how they were calculated

Our web collection on statistics for biologists contains articles on many of the points above.

Software and code

Policy information about availability of computer code

Data collection	ransmission electron microscopy (TEM) measurements were carried out on a Talos F200S transmission electron microscope (Thermo Fisher Scientific). A scanning electron microscope (SEM, SU-8010, Hitachi) was used to determine the morphology of the as-prepared samples. Powder X-ray diffraction patterns were recorded by Shimadzu XRD-7000 with Cu Kα radiation (λ = 1.5418 Å). High-angle annular dark-field scanning transmission electron microscopy (HAADF-STEM) characterization was conducted on an FEI Themis Z. Atomic force microscopy (AFM) images were taken using a Bruker Dimension ICON. XPS data were obtained with a K-Alpha electron spectrometer from Thermo Scientific. The actual Mn content was determined by using inductively coupled plasma optical emission spectroscopy (ICP-OES, Agilent 5110). The ζ potentials of as-prepared samples were measured by a Malvern Nano ZS90. Fourier Transform infrared spectroscopy (FTIR) spectra were obtained on a Thermo Scientific Nicolet iS20. Raman spectra were obtained on a Horiba LabRAM HR Evolution with an excitation wavelength of 532 nm. Ultraviolet-visible spectroscopy (UV-vis) measurements were recorded on a Shimadzu UV-2450 spectrophotometer. Fluorescence measurements were carried out using a Shimadzu RF-6000 spectrofluorometer. EPR data of radicals were obtained with a Bruker A200 and data of C3N4-Mn were obtained with a Bruker EMXplus-6/1. MS analysis was performed using an LTQ XL linear ion trap mass spectrometer (Thermo Fisher Scientific). High-resolution mass spectrum (HRMS) data were recorded on an AB triple-TOF 5600 HRMS. Confocal laser scanning microscopy (CLSM) images of cells were performed using a Laser Scanning Confocal Microscope system (Nikon A1). The X-ray absorption fine structure spectra were collected at the 1W1B station in the Beijing Synchrotron Radiation Facility (BSRF).
Data analysis	The XAS raw data were background subtracted, normalized, and Fourier transformed by standard procedures within the ATHENA program. The confocal fluorescence microscopy imaging data was analyzed by A1, Nikon (Japan). The MS data were analyzed by the Xcalibur software package. And also the ImageJ, Origin, Graphpad Prism.

For manuscripts utilizing custom algorithms or software that are central to the research but not yet described in published literature, software must be made available to editors and reviewers. We strongly encourage code deposition in a community repository (e.g. GitHub). See the Nature Portfolio guidelines for submitting code & software for further information.

Data

Policy information about availability of data

All manuscripts must include a data availability statement. This statement should provide the following information, where applicable:

- Accession codes, unique identifiers, or web links for publicly available datasets
- A description of any restrictions on data availability
- For clinical datasets or third party data, please ensure that the statement adheres to our policy

The authors declare that all data generated in this study are available within the article or the Supplementary Information. Other data related to this work are available from the corresponding authors upon request.

Research involving human participants, their data, or biological material

Policy information about studies with human participants or human data. See also policy information about sex, gender (identity/presentation), and sexual orientation and race, ethnicity and racism.

Reporting on sex and gender	N/A
Reporting on race, ethnicity, or other socially relevant groupings	N/A
Population characteristics	N/A
Recruitment	N/A
Ethics oversight	N/A

Note that full information on the approval of the study protocol must also be provided in the manuscript.

Field-specific reporting

Please select the one below that is the best fit for your research. If you are not sure, read the appropriate sections before making your selection.

☒ Life sciences ☐ Behavioural & social sciences ☐ Ecological, evolutionary & environmental sciences

For a reference copy of the document with all sections, see nature.com/documents/nr-reporting-summary-flat.pdf

Life sciences study design

All studies must disclose on these points even when the disclosure is negative.

Sample size	No statistical methods was used to predetermine the samples size. The sample sizes were determined as minimal to lower the cost and be sufficient to obtain statistically significant difference between experimental groups(n=3-7). For in vivo studies, each group contains 3 for evaluating the statistical significance. These sample sizes also represent the standard practice for publication in this field and were described in figure legends. Each sample represents independent biological replicates.
Data exclusions	No data were excluded from the analyses.
Replication	All experiments were repeated and experimental finding were reproducible.
Randomization	The cells and animals used in this paper were randomly distributed into several groups for the further experiments.
Blinding	The investigators were blinded to group allocation during experiments, data collection and analysis.

Reporting for specific materials, systems and methods

We require information from authors about some types of materials, experimental systems and methods used in many studies. Here, indicate whether each material, system or method listed is relevant to your study. If you are not sure if a list item applies to your research, read the appropriate section before selecting a response.

Materials & experimental systems

n/a	Involved in the study	
☒	☐	Antibodies
☐	☒	Eukaryotic cell lines
☒	☐	Palaeontology and archaeology
☐	☒	Animals and other organisms
☒	☐	Clinical data
☒	☐	Dual use research of concern
☒	☐	Plants

Methods

n/a	Involved in the study	
☒	☐	ChIP-seq
☒	☐	Flow cytometry
☒	☐	MRI-based neuroimaging

Eukaryotic cell lines

Policy information about cell lines and Sex and Gender in Research

Cell line source(s)	The human cervical cancer cells (HeLa cells) and the Human Umbilical Vein Endothelial Cells (HUV-EC cells) were purchased from The National Experimental Cell Resource Sharing Platform (NICR)
Authentication	These cells were authenticated by cell vitality test, mycoplasma detection and isozyme detection
Mycoplasma contamination	The cell line tested negative for mycoplasma contamination.
Commonly misidentified lines (See ICLAC register)	No commonly misidentified cell lines are used in this study.

Animals and other research organisms

Policy information about studies involving animals; ARRIVE guidelines recommended for reporting animal research, and Sex and Gender in Research

Laboratory animals	Female Balb/c mice (5 weeks) were purchased from Shanghai SLAC Laboratory Animal Co., Ltd.
Wild animals	The study did not involve wild animals.
Reporting on sex	Sex was not considered in the study.
Field-collected samples	The study did not involve samples collected from the field.
Ethics oversight	All animal experimental protocols were reviewed and approved by the Ethics Committee of Beijing Normal University and complied with all relevant ethical regulations (permit no. BNUCC-EAW-2023-16).

Note that full information on the approval of the study protocol must also be provided in the manuscript.

Plants

Seed stocks	N/A
Novel plant genotypes	N/A
Authentication	N/A
